# Fabrication of homotypic neural ribbons as a multiplex platform optimized for spinal cord delivery

**DOI:** 10.1038/s41598-020-69274-7

**Published:** 2020-07-31

**Authors:** Zachary T. Olmsted, Cinzia Stigliano, Abinaya Badri, Fuming Zhang, Asher Williams, Mattheos A. G. Koffas, Yubing Xie, Robert J. Linhardt, Jose Cibelli, Philip J. Horner, Janet L. Paluh

**Affiliations:** 1 Nanobioscience Constellation, Colleges of Nanoscale Science and Engineering, State University of New York Polytechnic Institute, NanoFab East, 257 Fuller Road, Albany, NY 12203 USA; 2 Center for Neuroregeneration, Department of Neurosurgery, Houston Methodist Research Institute, 6670 Bertner Ave. R10-North, Houston, TX 77030 USA; 3 Center for Biotechnology and Interdisciplinary Studies, Rensselaer Polytechnic Institute, 1623 15th St, Troy, NY 12180 USA; 4 Department of Animal Science, College of Agriculture and Natural Resources and Large Animal Clinical Sciences, College of Veterinary Medicine, Michigan State University, East Lansing, MI 48824 USA

**Keywords:** Bioinformatics, Biological models, Experimental organisms, Imaging, Microscopy, Biomaterials - cells, Implants, Cell adhesion, Cell death, Cell division, Cell signalling, Cellular imaging, Cytoskeleton, Glycobiology, Cell growth, Cell proliferation, Differentiation, Neurogenesis, Pattern formation, Pluripotency, Stem-cell niche, Stem cells, Experimental organisms, Model vertebrates, Neural stem cells, Stem-cell differentiation, Cell death in the nervous system, Glial biology, Neuroimmunology, Peripheral nervous system, Stem cells in the nervous system, Biological techniques, Biotechnology, Cell biology, Developmental biology, Neuroscience, Stem cells, Chemical biology, Enzymes, Glycobiology

## Abstract

Cell therapy for the injured spinal cord will rely on combined advances in human stem cell technologies and delivery strategies. Here we encapsulate homotypic spinal cord neural stem cells (scNSCs) in an alginate-based neural ribbon delivery platform. We perform a comprehensive in vitro analysis and qualitatively demonstrate graft survival and injury site retention using a rat C4 hemi-contusion model. Pre-configured neural ribbons are transport-stable modules that enable site-ready injection, and can support scNSC survival and retention in vivo. Neural ribbons offer multifunctionality in vitro including co-encapsulation of the injury site extracellular matrix modifier chondroitinase ABC (chABC), tested here in glial scar models, and ability of cervically-patterned scNSCs to differentiate within neural ribbons and project axons for integration with 3-D external matrices. This is the first extensive in vitro characterization of neural ribbon technology, and constitutes a plausible method for reproducible delivery, placement, and retention of viable neural cells in vivo.

## Introduction

The adaptability of transplanted neural stem cells (NSCs) to replace damaged or malfunctioning cell types through differentiation and integration in vivo has justified their use in myriad central nervous system (CNS) disorders such as neurodegeneration^1^, trauma^2^, and stroke^3^. In traumatic spinal cord injury (SCI), acute and chronic damage is sustained to multiple components of the CNS cytoarchitecture, including partial or complete loss of neural circuitry^4^. To date, delivery of unprotected NSCs in suspension has produced mixed results for functional improvement in SCI. Refinements in choice of NSCs in SCI cell therapies are now considering neuroanatomical and cellular diversity along the rostral–caudal and dorsal–ventral axes^5^. Such natural physiological complexity requires development of well-defined and reproducible differentiation protocols to generate regionally-specified NSCs corresponding to the level of injury that are currently lacking. It has recently been demonstrated that rapid and successful integration into grafts requires that functional restrictions be overcome by homotypic matching with tissue-specific targets^6^. This is emphasized by one study in the SCI field using stem cell-derived NSCs with general spinal cord identity versus forebrain identity^5^, and similarly in Parkinson’s disease cell therapy by a shift towards A9 midbrain dopaminergic progenitors^7^. Such region-specific cell choice strategies are providing hope that barriers to survival and integration of transplanted cells in the injured spinal cord are addressable. However, equally important is the ability to deliver reproducibly differentiated cells in neuroprotective platforms^8^ .

A wave of inflammatory responses in SCI challenges retention and viability of therapeutic cells transplanted without use of a delivery platform^8^. Freely delivered cells lack a guide for positioning and retention at the injury site^9^ and for retaining modulators co-delivered to address the hostile injury cavity such as those being explored to remove extracellular matrix (ECM) inhibitory signals^10, 11^. Encapsulation of cells may offer the most broadly applicable therapeutic delivery platform to allow refinement of additional parameters including cell type(s), cell source, cell developmental maturation and functional state, cell dose, and the timing of intervention^12^. Currently, no uniform delivery platform exists, and this gap limits reproducibility that would enable refinement of such parameters for SCI across animal studies. Therefore, advancing and refining delivery platforms is of utmost importance to improve interpretation of in vivo studies and to assist the field in translating confidently to the clinic. Rigorous comparison and optimization of cell-based therapies therefore requires focused and comprehensive efforts in designing reproducible, multipurpose platforms for cell transplantation to address these challenges.

Here we report a first-of-a-kind method for the generation, handling, imaging, and CNS in vivo delivery of homotypic neural ribbons. By refining and advancing use of alginate-based hydrogels and generation of homotypic hiPSC-derived spinal cord-enhanced NSCs (scNSCs) we combine two critical technologies. We apply spinal cord developmental signaling using neuromesodermal progenitor (NMp) intermediates to generate cervically-patterned scNSCs and extensively characterize these cells. In contrast to traditional iPSC protocols that produce NSCs first with forebrain identity^13,14^, NMps are the natural origin of the spinal cord and adjacent mesodermal somites^15^. The homotypic scNSCs encapsulated in neural ribbons retain robust viability and multipotency, and extend axons orthogonally to integrate into 3-D ECM test matrices. We further demonstrate neural ribbon multifunctionality by inhibitory microenvironment modification using co-encapsulated enzyme chABC. The typical cell dose for rat models of SCI ranges from 200,000 to 2 million cells, and is performed on-site with cells prepared immediately prior to transplantation. Using our protective neural ribbon strategy, we demonstrate qualitative cell survival within the contused injury cavity using a cell number as low as ~ 5,000, whereas delivery of unprotected cells was less robust. Neural ribbons are durable, and can be prepared at one location and shipped off-site for animal studies. We apply a well-established rat C4 hemi-contusion injury model^16^ to validate neural ribbon delivery by syringe injection. Homotypic cervical scNSCs from neural ribbon grafts survive and are retained in the injury cavity, projecting neurites into host tissue that co-localize with Synapsin. In summary, multiplex neural ribbons are neuroprotective, transport-stable modules that support delivery of homotypic neural cells to the CNS. This customizable strategy provides an opportunity to continue to unify exciting research in the SCI field by many laboratories towards a common goal. That is, to overcome in vivo barriers and expedite bench-to-bedside clinical translation and efficacy.

## Results

### hiPSC-derived homotypic NSCs exhibit cervical spinal cord identity

**Figure 1:**
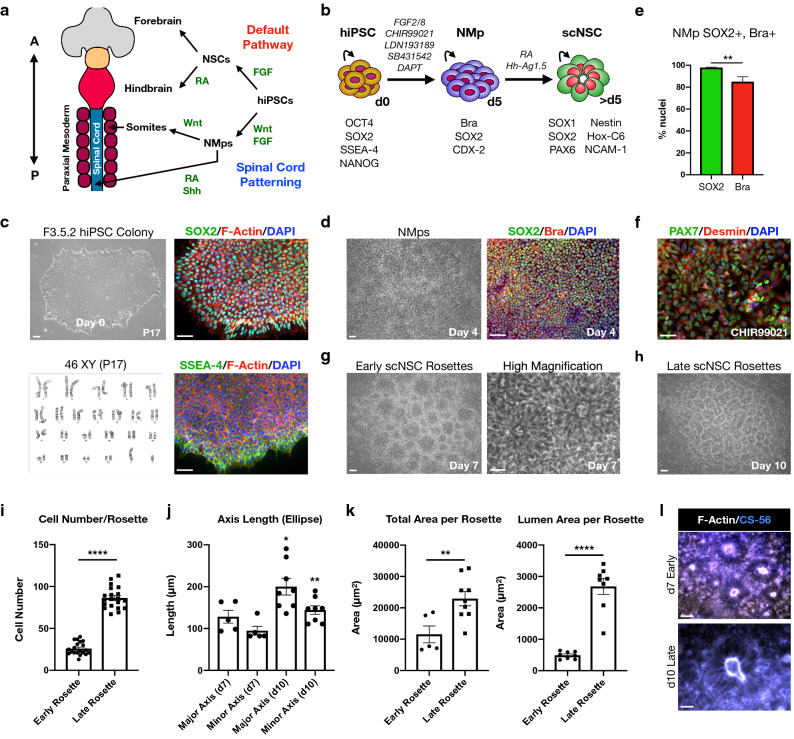
Generation of hiPSC-derived NMps and homotypic NSC rosettes at high-efficiency. (**a**) Diagram for NSC patterning using the default anterior (A) or spinal cord posterior (P) pathway that are developmentally distinct. (**b**) Differentiation protocol used to generate scNSC rosettes from hiPSCs through an NMp intermediate stage. (**c**) F3.5.2 hiPSC line used for scNSC differentiation. Top (left-to-right): phase contrast image of F3.5.2 hiPSC colony; SOX2/F-Actin. Bottom (left-to-right): hiPSC passage 17 normal karyotype; SSEA-4/F-Actin, nuclei stained with DAPI. (**d**) Day 4 NMps (left) and co-localization of SOX2/Brachyury (Bra). (**e**) Histogram of SOX2 (97.9 ± 0.4%) and Bra (85.0 ± 4.5%) as percent of nuclei, day 4. n = 3 fields averaged, N = 669 cells. (two-tailed t test, t = 4.981, df = 4, **P = 0.0076). Data reported as (mean ± s.e.m.). (**f**) PAX7/Desmin myoblasts. (**g**) scNSC early-stage rosettes (day 7, left); high-magnification rosettes (right). (**h**) Late stage rosettes (day 10). (**i**) Histogram of cell number per rosette for early- (26 ± 2 cells/early rosette) and late-stage (86 ± 3 cells/late rosette) time-points. (two-tailed t test, t = 18.1, df = 38, ****P < 0.0001, n = 20 rosettes each). (**j**) Histogram of major and minor axis length of early- (128 ± 15 µm; 95 ± 11 µm) and late-stage (200 ± 20 µm; 144 ± 10 µm) rosettes approximated as ellipses. Major axes (two-tailed t test, t = 2.5, df = 11, *P < 0.0271); minor axes (two-tailed t test, t = 3.17, df = 11, **P < 0.009); n = 5 early-, n = 8 late-stage. (**k**) Left: histogram of total area per rosette for early- (11,500 ± 2,400 µm^2^) and late-stage (22,900 ± 2,300 µm^2^) rosettes. (two-tailed t test, t = 3.12, df = 12, **P < 0.009). n = 5 for early-, n = 9 for late-stage. Right: histogram of lumen area per rosette for early- (490 ± 41 µm^2^) and late-stage (2,700 ± 250 µm^2^). (two-tailed t test, t = 8.56, df = 14, ****P < 0.0001). n = 8 rosettes per condition. (**l**) Early- versus late-stage scNSC rosettes stained with F-Actin and CS-56.

NSC production from pluripotent stem cells by default yields anterior directed cells with forebrain identity (Fig. 1a)^13,14^. To generate NSCs with spinal cord identity, we first induced bipotent NMp intermediates (Fig. 1b) by targeting Wnt and FGF signaling using established methods^5,17,18^, with the well-characterized hiPSC line F3.5.2 developed in our laboratory (Fig. 1c–e)^19,20^. The GSK-3β inhibitor small molecule CHIR99021 and FGF2/FGF8 recombinant proteins were used to induce SOX2+/Brachyury+ NMps. Simultaneously, induction efficiency was increased by dual SMAD inhibition^21,22^ using LDN193189 (BMP inhibitor) and SB431542 (Activin, TGF-β inhibitor) in combination with DAPT (γ-secretase inhibitor)^23^ that together prevent endodermal and mesodermal differentiation. After 3–4 days of induction, colonies were morphologically distinct from hiPSCs (Fig. 1c,d) with robust co-expression of SOX2 and Bra proteins (Fig. 1d,e). At day 4, 97.9 ± 0.4% of cells (mean ± s.e.m.) expressed SOX2 and 85.0 ± 4.5% expressed Bra as percentage of total nuclei (Fig. 1e; two-tailed t test, t = 4.981, df = 4, **P = 0.0076; three fields were averaged for a total of N = 669 cells). By maintaining NMps in N2B27 basal medium supplemented with CHIR99021 only, we generated PAX7+/Desmin+ myoblasts (Fig. 1f) demonstrating NMp ability to produce mesodermal phenotypes. These data support the rapid and efficient production of NMps from hiPSCs, resulting in a progenitor state primed for the generation of spinal cord enhanced NSCs (scNSCs).

scNSCs were cervically-patterned by transitioning NMps from defined Wnt/FGF exposure to the caudalizing morphogen retinoic acid (RA; 100 nM) at day 5 in differentiation (Fig. 1b)^24,25^. We also induced ventralization of scNSCs to the motor neuron progenitor domain (pMN) of the dorsal–ventral axis by adding the Sonic hedgehog (Shh) agonist Hh-Ag1.5 (200 nM) at the same time that RA was added. The combination of RA and Hh-Ag1.5 was sufficient to induce rosette formation within two days. Rosette size and cell number was determined to inform on the ideal scNSC stage and differentiation time point for incorporation into neural ribbons. By day 7, early-stage rosettes had distinct lumens, albeit they were smaller and contained fewer cells when compared to day 10 rosettes (Fig. 1g,h). Rosette cell number was evaluated by immunofluorescence (IF) with NSC marker SOX2. On average, late-stage rosettes contain ~ 3.3-fold more cells than early-stage rosettes (Fig. 1i; 26 ± 2 cells/early rosette vs. 86 ± 3 cells/late rosette; ****P < 0.0001). Major and minor axes of late rosettes were significantly greater than early rosettes (Fig. 1j; major axis: 200 ± 20  µm late vs. 128 ± 15 µm early, *P < 0.0271; minor axis 144 ± 10 µm late vs. 95 ± 11 µm early, **P < 0.009). The rosette total area (22,900 ± 2,300 µm^2^ late vs. 11,500 ± 2,400 µm^2^ early) and lumen area (2,700 ± 250 µm^2^ vs. 493 ± 41 µm^2^) calculated directly from ROI were also increased (Fig. 1k) but scaled differently during maturation. Notably, the increase in total rosette area from day 7 to day 10 scaled by a factor of ~ 2x (**P < 0.009), while the increase in lumen area scaled by a factor of ~ 6.25x (****P < 0.0001; detailed in Fig. 1 legend). Early- and late-stage rosettes were also distinguished by CS-56 antibody specific to glycosaminoglycan (GAG) chains of native chondroitin sulfate proteoglycans (CSPGs; Fig. 1l).

**Figure 2:**
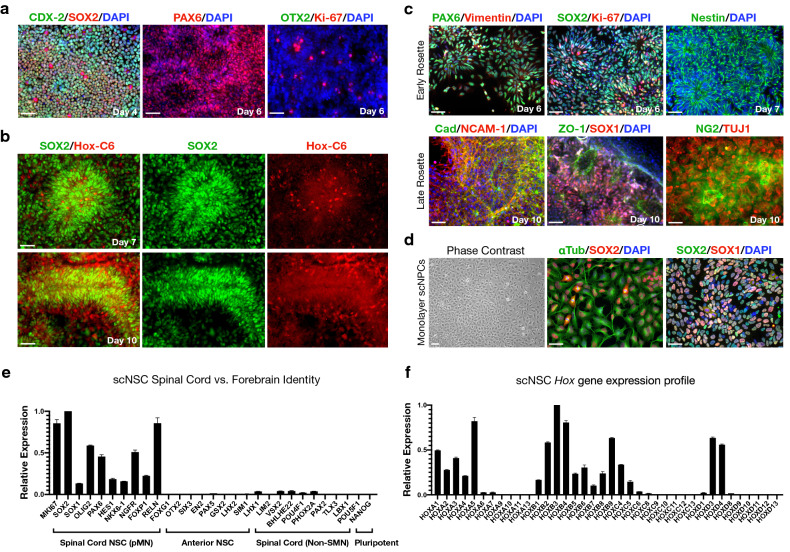
NSCs have spinal cord and not forebrain identity. (**a**) Left-to-right: CDX-2/SOX2 NMps (day 4); PAX6+ rosettes (day 6); forebrain-specific OTX2 negative control co-stained with Ki-67 (day 6). (**b**) Localization of cervical spinal cord protein, Hox-C6, and SOX2 (top: day 7; bottom: day 10). (**c**) NSC biomarker expression in differentiating scNSCs. Top (early-stage rosettes; left-to-right): PAX6/Vimentin (day 6); SOX2/Ki-67 (day 6); Nestin (day 7). Bottom (late-stage rosettes, day 10; left-to-right): Pan-Cadherin/NCAM-1/DAPI; ZO-1/SOX1/DAPI; NG2/TUJ1. (**d**) Dissociation of rosettes as spinal cord neural progenitor cells (left-to-right): phase contrast; α-Tubulin/SOX2/DAPI; SOX2/SOX1/DAPI. (**e**) Relative gene expression of day 10 scNSC rosettes using RNA-Seq normalized counts (mean ± s.e.m., N = 2 differentiations) to interrogate distinct neural populations and pluripotency genes. (**f**) scNSC *Hox* gene expression profile. Data were normalized to the highest value for each group that was set to 1. Scale bars are 50 µm.

By day 4 of NMp induction, nearly 100% of cells expressing SOX2 co-expressed caudal type homeobox protein 2 (CDX-2), the upstream master regulator of posterior *Hox* genes^26^. One day after transitioning to RA/Hh-Ag1.5-mediated patterning, expression of NSC transcription factor paired box 6 (PAX6) was substantially upregulated and the forebrain NSC marker OTX2 was not detected (Fig. 2a). Additionally, cervical/brachial homeobox transcription factor, Hox-C6, was expressed in both early- and late-stage rosettes (Fig. 2b). Early rosettes were immunopositive for NSC intermediate filament proteins Vimentin and Nestin, as well as Ki-67 indicating actively proliferating cells (Fig. 2c, top). Late-stage rosettes characteristically expressed cadherin and neural cell adhesion molecule 1 (NCAM-1), the NSC transcription factor SOX1, and exhibited redistribution of tight junction protein ZO-1 apically to the rosette lumen. Committed TUJ1+ neuronal progenitors extended outwards from the basal surface of rosettes that were immunopositive for the NG2 proteoglycan (Fig. 2c, bottom). Manual isolation and re-plating of rosettes onto fresh substrates yielded monolayer spinal cord neural progenitor cell cultures retaining SOX1/SOX2 positivity and cell-cycling ability (Fig. 2d). Day 10 scNSC rosettes robustly express genes characteristic of the spinal cord pMN domain but not anterior/forebrain development or pluripotency by RNA-Seq. Genes identifying distinct neuronal progenitor populations of the spinal cord other than MNPs are expressed at low levels (Fig. 2e). Also expressed are a suite of rostral *Hox* genes, corroborating cervical/brachial spinal cord identity (Fig. 2f).

### scNSCs differentiate into posterior CNS cells in vitro

**Figure 3:**
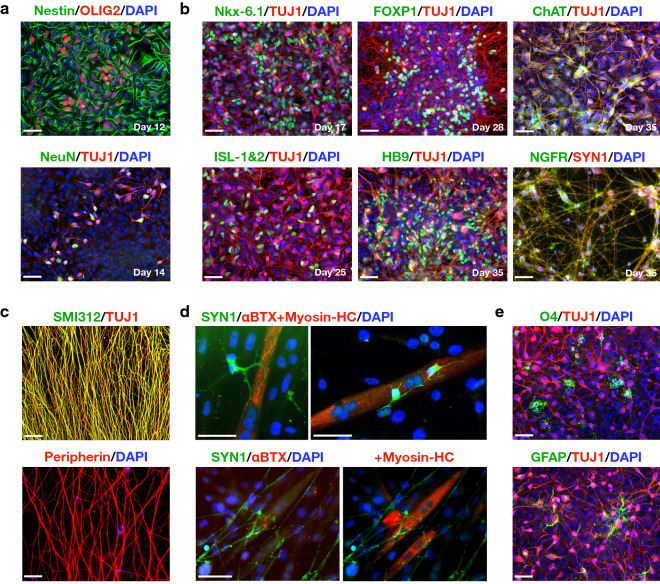
scNSCs differentiate into committed MNPs and SMNs. (**a**) Caudal-ventral scNSCs generate motor neuron progenitors (MNPs): Nestin/OLIG2 (day 12, top); NeuN/TUJ1 (day 14, bottom). Cells are counterstained with DAPI (nuclei). (**b**) Cervically patterned scNSCs yield primarily cholinergic limb-innervating SMNs. Top (left-to-right): IF image of pMN TF Nkx-6.1 (day 17); limb-innervating lateral motor column TF FOXP1 (day 28); choline acetyltransferase, ChAT (day 35). Bottom (left-to-right): SMN-related TF ISL-1&2 (day 25); SMN-specific TF HB9 (day 35); nerve growth factor receptor NGFR (day 35). Cells are counterstained with TUJ1 or SYN1 and DAPI. (**c**) Axon identification using pan-axonal biomarker SMI312 (high MWT phosphorylated neurofilaments) counterstained with TUJ1 (top); PNS-specific intermediate filament protein Peripherin (bottom). (**d**) Cholinergic SMNs preferentially interact with multinucleated myotubes and assemble NMJ machinery. Top images: Synapsin 1/αBTX-594 + Myosin Heavy Chain (Myosin-HC). Bottom images: SYN1 (pre-synaptic terminals; green)/αBTX-594 (post-synaptic nAChR clusters) co-localization supports early NMJ formation (left). Samples were subsequently stained with Myosin-HC to validate skeletal myotubes (right). (**e**) Identification of two additional CNS cell types in differentiating SMN cultures supports scNSC multipotency (day 42). Top: OPCs (O4/TUJ1); bottom: astrocytes (GFAP/TUJ1). Scale bars are 50 µm.

Under continued exposure to RA/Hh-Ag1.5, differentiating cultures expressed pMN-specific transcription factor OLIG2 by day 12 (Fig. 3a). By day 14, the neuronal nuclear antigen NeuN was observed to co-localize in TUJ1+ cells, suggesting an early stage of motor neuron progenitor. From this point, robust neurogenesis was observed, and differentiating neuronal cultures developed a step-wise biomarker profile characteristic of cervical/brachial SMNs (Fig. 3b)^27^. That is, the pMN-specific transcription factor Nkx-6.1, terminal SMN-related ISL-1&2, lateral motor column protein FOXP1, terminal SMN-specific HB9/*MNX1*, cholinergic neurotransmitter enzyme ChAT, and the nerve growth factor receptor NGFR/CD271/p75NTR. SMN maturation is supported by phosphorylation of high molecular weight (MWT) neurofilaments in axons using the SMI312 antibody cocktail (Fig. 3c), and Peripherin, an intermediate filament marker of the peripheral nervous system. We observed that hiPSC-derived SMNs show high affinity for rodent myotubes and assemble neuromuscular junctions (NMJs) in co-cultures (Fig. 3d) as assessed by co-localization of Synapsin 1 (SYN1; pre-synaptic terminals) with α-Bungarotoxin (αBTX) targeting nicotinic acetylcholine receptors (nAChR, post-synaptic NMJ terminal). We also identified O4+ oligodendrocyte progenitor cells (OPCs) and GFAP+ astrocytes in differentiating cultures (Fig. 3e), validating scNSC ability to produce multiple cell types of the spinal cord.

### Co-encapsulation of scNSCs and chABC in multiplex neural ribbons

**Figure 4:**
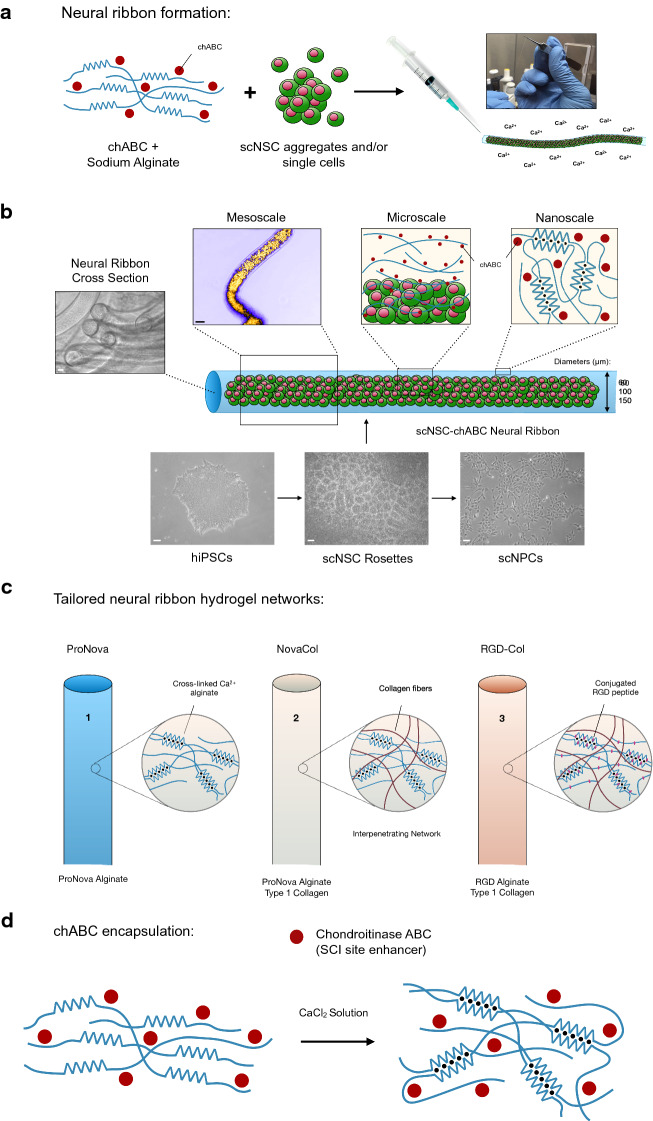
Overview of scNSC and chABC co-encapsulation in neural ribbons. (**a**) Cartoon diagram of alginate ribbon system used to co-encapsulate scNSCs with chABC. (**b**) Multiscale organization of alginate ribbons containing co-encapsulated scNSCs and chABC. Cross-sections of alginate ribbons demonstrate cylindrical character. (**c**) Composition of ribbons used in this study shown with cartoons of nanoscale structure. (**d**) Physical entrapment of chABC (red circles; 115 kDa) by alginate chains during gelation.

We established an alginate hydrogel-based neural ribbon system for rapid, reproducible encapsulation of scNSCs and co-encapsulation with the microenvironment regulator chondroitinase ABC (chABC; Fig. 4). Alginate chains crosslink via aggregation of guluronate blocks in the presence of divalent Ca^2+^ ions allowing moldability into 3-D hydrogel networks^28^. In our system, chABC and scNSCs were combined with 1.5% sodium alginate and co-encapsulated by constrained needle tip extrusion into 100 mM CaCl_2_ (Fig. 4a,b,d). Modified hydrogel ribbons were also investigated on the basis of interpenetrating network (IPN) formation with collagen to provide exogenous ECM and RGD-functionalized bioactive peptides for improved cell–cell interactions. We generated three separate ribbon compositions to achieve goal-directed structural and functional outcomes (Fig. 4c) that are ultrapure sodium alginate (ProNova) alone, ProNova alginate-collagen IPNs (NovaCol) and RGD-functionalized alginate-collagen IPNs (RGD-Col; Supplementary Table 3, 4).

Hydrogel ribbons with specified cross-sectional area were templated by needle tip inner diameter. Three diameters were compared (60, 100, 150 µm) that are suitable for injection into a hemi-contusion-induced SCI cavity in rat models, and that each permit access of gases, nutrients and extracellular signals to cells at the neural ribbon core^29,30^. To enable ribbon visualization for transplantation studies, co-encapsulation of fluorescent indicators was done and signal retention monitored (Supplementary Fig. 1). Biodegradation of alginate hydrogel was tested by exposure to artificial cerebrospinal fluid (aCSF) with daily solution changes and determination of the ratio of gel wet weight remaining with respect to initial formation. Bulk alginate was reduced to ~ 50% by day 7 and to 11% by day 21. A parallel experiment using 150 µm ribbons evaluated mechanical resistance to weight of a glass coverslip at similar corresponding time points. At day 21, ribbons did not withstand cover slip addition and degraded hydrogel was visible (Supplementary Fig. 1).

### Hydrogel ribbons containing chABC regulate external CSPG ECM

**Figure 5:**
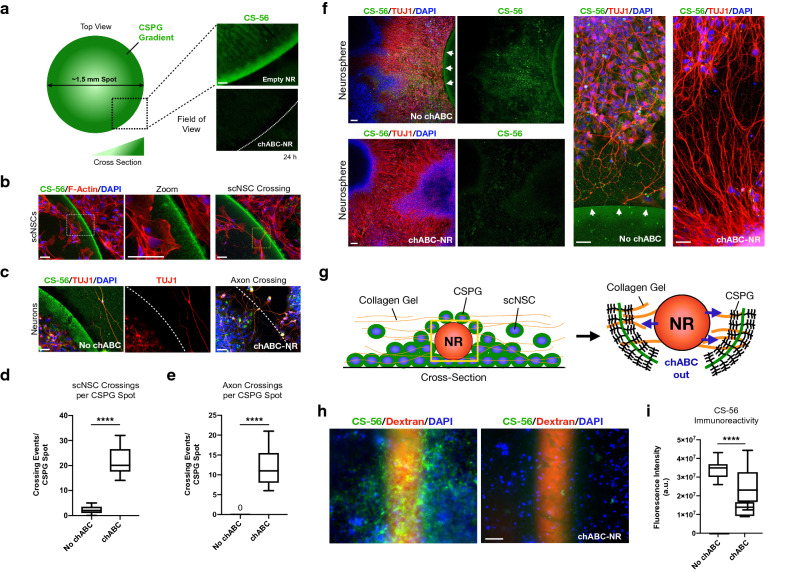
Favorable CSPG microenvironment transformation by neural ribbon-encapsulated chABC. (**a**) In vitro CSPG glial scar model. Left: schematic of in vitro CSPG spot assay. Right: CSPG gradient visualized by CS-56 (green) incubated for 24 h in aCSF with empty ribbon (top) or chABC-encapsulated neural ribbon (chABC-NR; bottom). CSPG rim indicated by white dotted line. (**b**) CSPG gradient (CS-56) excludes scNSCs (F-Actin/DAPI; leftmost image; zoomed image, left-center). Occasionally, scNSCs penetrate and cross over without chABC treatment (yellow dotted box; rightmost image). (**c**) chABC-NR enables axon navigation of the inhibitory space. Left: axon exclusion by CSPG gradient. Right: chABC-encapsulated ribbon incubation increases axon crossing events. CS-56/TUJ1/DAPI. (**d**) Box and whisker plot of scNSC crossings per CSPG spot +/− chABC-NR. (two-tailed t test, t = 9.87, df = 16, ****P < 0.0001). n = 9 CSPG spots per condition. (**e**) Box-and-whisker plot of axon crossings per CSPG spot +/− chABC-NR. (two-tailed t test, t = 7.683, df = 8, ****P < 0.0001). n = 9 CSPG spots per condition. (**f**) CSPG deterrence of axon outgrowth from SMN neurospheres +/− chABC-NR. No chABC (top-left) versus chABC-NR incubation (bottom-left) with high-magnification images (right). White arrows indicate spot edge. (**g**) Cartoon overview of experiment in (**h**). chABC-NR (red), CSPG-secreting scNSCs (green, blue, respectively) and collagen immobilizing matrix (orange fibers) are labeled. (**h**) chABC release from ribbons attenuates endogenous CSPG production by scNSCs. No chABC (left) versus chABC-NR incubation (right). CS-56/Dextran/DAPI. (**i**) Box-and-whisker plot of CS-56 immunoreactivity from (**h**). (two-tailed t test, t = 9.63, df = 14, ****P < 0.0001). n = 9 fields averaged (no chABC), n = 7 fields averaged (chABC-NR). Scale bars are 50 µm.

To incorporate chABC encapsulation and delivery capability into our neural ribbon technology, we used in-house purified recombinant *P. vulgaris* chABC enzyme generated using two bacterial systems that are gram-negative *E. coli* and gram-positive *Bacillus megaterium*. By UV–Vis digestion assays with chondroitin sulfate (CS) substrates, both purified enzymes performed as well as the standard commercial chABC product (Supplementary Table 7). We focused on *Bacillus*-purified recombinant chABC that is endotoxin-free for further analysis to extend chABC to animal studies. chABC activity was tested using multiple in vitro assays exploiting the endogenous production of CSPGs by scNSCs (Supplementary Fig. 2) as well as acellular CSPG gradient spot assays for glial scar modeling (Fig. 5)^31,32^. Despite the endogenous production of CSPG ECM, scNSCs are largely prohibited from penetrating the concentrated rim of CSPG inverse gradient spots. The CS-56 antibody enables visualization of both the CSPG spot and punctate intracellular CSPG signal. In the presence of chABC, CSPG signal is restricted to the perinuclear region of neural cells but does not impact scNSC proliferation or viability. Notably, expression of the functional inhibitory CSPG receptor LAR and PTPσ are similarly expressed during differentiation from scNSC to SMN (Supplementary Fig. 2).

We investigated in vitro modulation of the scNSC CSPG microenvironment by encapsulated release of chABC from neural ribbons (Fig. 5; chABC-NR). CSPG inverse gradient spots were formed to mimic the glial scar, as previously described^31,32^. CSPG spots were completely degraded by 24 h after incubation with chABC-NR (0.1 U/ml working concentration) in aCSF (Fig. 5a). By seeding scNSCs onto CSPG spots, we observed that the inverse concentration gradient is inhibitory to scNSCs despite endogenous CSPG production. Occasionally we observed a spontaneous cell-crossing event (~ 2 events/spot). The frequency of such events was significantly increased by incubation with chABC-NR (~ 22 events/spot; Fig. 5b, d; two-tailed t test, t = 9.87, df = 16, ****P < 0.0001; N = 9 CSPG spots counted per condition). Seeding of scNSC-derived SMNs onto CSPG spots also revealed a marked inability of axons to penetrate the CSPG inhibitory rim, while incubation with chABC-NR enabled axon migration and navigation within this region (Fig. 5c). Again, axon crossing events were significantly increased from 0 to ~ 12 events/spot by addition of chABC-NR (Fig. 5e; t = 7.68, df = 8, ****P < 0.0001; N = 9 CSPG spots counted per condition). This effect of chABC-NR was replicated using plated SMN neurospheres that undergo robust axonal outgrowth (Fig. 5f). Finally, we investigated the ability of encapsulated chABC to modulate the inhibitory CSPG microenvironment using an scNSC-based assay (Fig. 5g–i). Single Dextran-labeled chABC-NR were incubated with confluent scNSC cultures and embedded in Type 1 collagen matrix for immobilization (Fig. 5g). As a negative control for microenvironment modulation, the chABC-NR condition was compared to ribbons without chABC (Fig. 5h). chABC-NR co-culture resulted in a dramatic attenuation of extracellular scNSC CSPGs (0.1 U/ml working concentration) versus the negative control, and was quantified by CS-56 fluorescence intensity (Fig. 5i; t = 9.63, df = 14, ****P < 0.0001).

### Homotypic scNSCs survive and retain differentiation potential in neural ribbons

**Figure 6:**
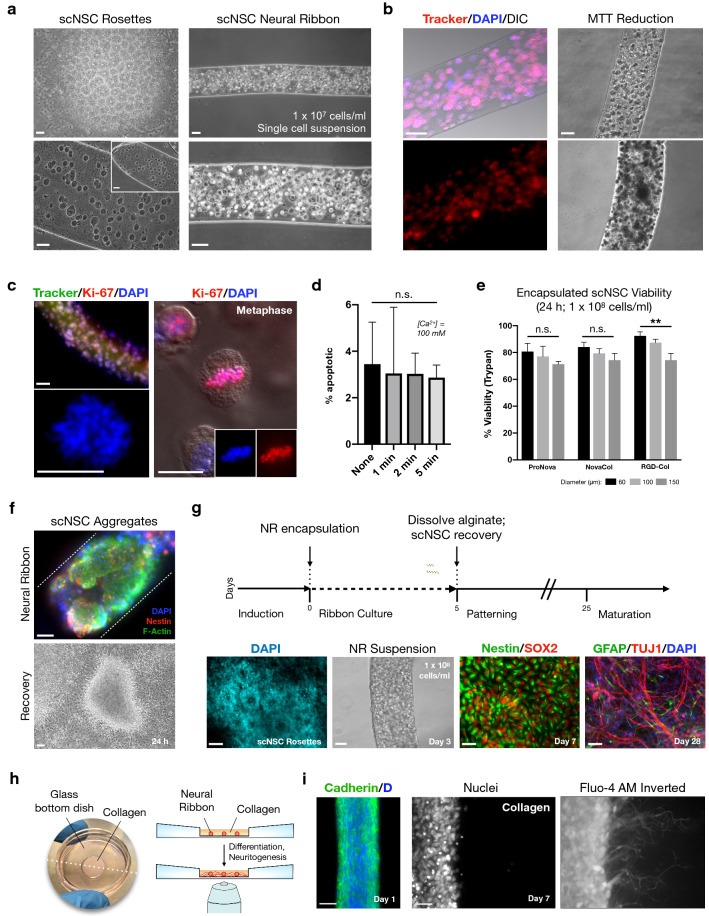
Neural ribbons maintain scNSC viability, cell cycling and differentiation potential. (**a**) Clockwise: Dissociated scNSC rosettes encapsulated in 1.5% alginate ribbons (1 × 10^7^ cells/ml); zoom image; transfer to N2B27 medium. Inset is ribbon end. (**b**) Metabolic activity. Left: live neural ribbons imaged 24 h after loading (1 × 10^7^ cells/ml). CellTracker scNSCs counterstained with DAPI. DIC and merged images shown. Right: neural ribbon before (top) and after (bottom) MTT reduction to insoluble formazan. (**c**) scNSCs in neural ribbons retain cell cycle ability. Top-left: CellTracker/Ki-67/DAPI. Bottom-left: mitotic figure in neural ribbons (DAPI). Right: mitotic figure co-stained with Ki-67. (**d**) Histogram quantification of percent apoptotic cells for no exposure or t = 1, 2 and 5 min exposure to 100 mM CaCl_2_. (one-way ANOVA, F = 0.05818, R^2^ = 0.02135, n.s. *P* = 0.98). N > 500 cells counted per condition (average three separate fields per condition). (**e**) scNSCs viability at 24 h by Trypan blue exclusion (1 × 10^8^ cells/ml). Three compositions tested at three diameters (60, 100, 150 µm). (RGD-Col one-way ANOVA, F = 19.6, **P = 0.002). n = 300 cells from three ribbons counted per condition. (**f**) Aggregate loading improves scNSC viability and recovery in neural ribbons. Top: IF image of F-Actin/Nestin/DAPI. White dotted lines indicate ribbon edge. Bottom: scNSC aggregates recovered from ribbons. (**g**) Neural ribbon scNSCs retain multipotency. Top: experimental overview. Bottom: scNSC rosettes (DAPI; leftmost); scNSC-NR suspension culture (day 3; left-center); recovered scNSCs (day 7, Nestin/SOX2; right-center); differentiation (day 28, GFAP/TUJ1/DAPI; rightmost). scNSCs from three replicate neural ribbons were pooled to seed each coverslip. Three repeat experiments performed. (**h**,**i**) Neural ribbons project neurites and integrate into 3-D ECM matrices. (**h**) White light image (left) and overview (right) of neural ribbon immobilization platform. Neural ribbon (red) is immobilized in collagen matrix (orange). (**i**) Pan-Cadherin/DAPI day 1 post-encapsulation (leftmost); Live-cell calcium imaging (Fluo-4 AM/DAPI) of scNSCs within NovaCol ribbons with lateral neurite outgrowth (day 7 post-encapsulation). Inverted LUT is shown (right image). Day 7 average neurite length 223 ± 34 µm (n = 54 neurites measured from N = 6 fields). Data reported as (mean ± s.e.m.). Scale bars are 50 µm except for high magnification images in (**c**) that are 10 µm.

We confirmed and optimized the biocompatibility of hydrogel ribbons with scNSCs in terms of cell viability, proliferation, and multipotent differentiation potential (Fig. 6). Dissociation of day 10 scNSC rosettes into single cell suspensions and subsequent encapsulation (1 × 10^7^ cells/ml) yields a disperse distribution of cells within the ribbons, particularly at 150 µm diameter (Fig. 6a). The encapsulated scNSCs retained viability (CellTracker) and metabolic activity (MTT assay) at 24 h (Fig. 6b), and dividing cells with mitotic figures were observed by DAPI and the Ki-67 antibody (Fig. 6c). A live-cell dual apoptosis reporter kit (488 NucView Caspase-3 substrate co-localization with TexasRed-Annexin V) was used to quantify apoptotic cells in adherent scNSC cultures (day 10) exposed to 100 mM CaCl_2_ for durations relevant to ribbon initial formation and handling (no exposure, 1, 2, 5 min). The percentage of apoptotic cells was slightly reduced over time, although insignificantly (Fig. 6d; one-way ANOVA, F = 0.058, R2 = 0.021, n.s. *P* = 0.98; n > 500 total cells counted per condition, average of three separate fields per condition). A Ca^2+^-mediated lift-off process may disproportionally affect loosely bound apoptotic cells. We next analyzed the effect of ribbon diameter on viability using Trypan blue exclusion on cells recovered from 60, 100 and 150 µm neural ribbons. Single cell suspension scNSCs encapsulated at high density (1 × 10^8^ cells/ml; day 10) were recovered 24 h post-encapsulation by dissolving alginate ribbons in 1.6% sodium citrate (Fig. 6e; see also Supplementary Table 3). RGD-Col neural ribbons yielded the highest viability of scNSCs, presumably due to improved cell adhesion and migration, but varied with diameter (one-way ANOVA, F = 19.6, **P = 0.0024; n = 300 cells from three ribbons analyzed per condition). Viability remained high in ProNova and NovaCol ribbons, but with no significant variation within groups by one-way ANOVA. Viability increased with decreasing ribbon diameter independent of ribbon composition. Using the higher cell loading concentration, neural ribbon quality and cell health improved overall. As well, loading of neural aggregates (~ 50–100 µm) that retain cell–cell interactions (Fig. 6f) provided superior results compared to single cell suspensions. Easy handling in culture medium allows for efficient transfer of neural ribbons to cryovials for long distance shipping (> 1,750 miles; 20–24 h laboratory-to-laboratory) in a portable incubator (Supplementary Fig. 1) enabling offsite in vivo studies. Finally, neural ribbons (1 × 10^8^ cells/ml) cultured in suspension for five days in scNSC maintenance medium^5^ retain multipotency and produce TUJ1+ neurons and GFAP+ astrocytes (Fig. 6g).

### Neural ribbons extend neurites for 3-D matrix microenvironment integration

We tested the ability of scNSCs to differentiate within NovaCol ribbons and integrate into external collagen matrices in vitro (Fig. 6h, i). We developed a neural ribbon analysis platform by immobilization in collagen gel in the viewing area of a glass bottom dish (Fig. 6h). This strategy optimizes imaging efficiency and statistical analysis. Encapsulated scNSCs were visualized by live cell imaging with Fluo-4 AM (NovaCol, 60 µm diameter), along with Cadherin IF (Fig. 6i). Incubation of the neural ribbon in CPM drives differentiation to MNPs and neuritogenesis. Robust lateral extension of neurites is visible by day 7 after encapsulation, extending orthogonally out of the ribbon body and into the surrounding collagen matrix (Fig. 6i). Average neurite length at day 7 as measured from the edge of the ribbon using projected 2-D images was 223 ± 34 µm (n = 54 neurites from N = 6 fields). Pan-Cadherin antibody staining reveals maintenance of extensive calcium-dependent cell–cell connections within the NovaCol hydrogel network even as early as day 1 after extrusion.

### Neural ribbon low-dose scNSC survival and injury site integration in vivo

**Figure 7:**
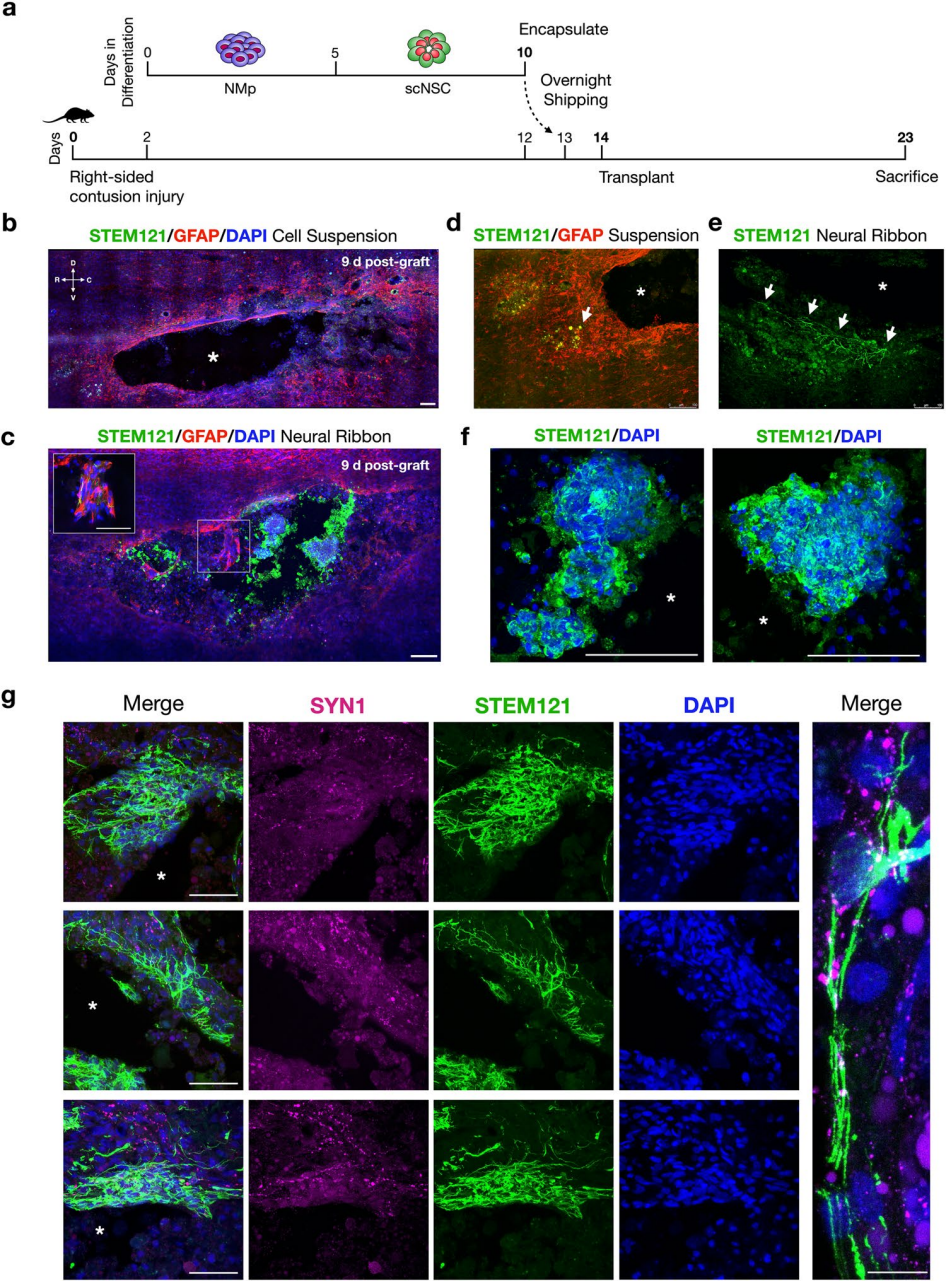
Injectable neural ribbons assist homotypic scNSC survival and retention in the contused injury cavity. (**a**) Choreographed timeline of pilot in vivo study relating stem cell differentiation and overnight shipping (Albany, NY) to C4 hemi-contusion injury and transplantation (Houston, TX). (**b**) scNSCs injected in suspension outside of neural ribbons are scarcely detected. Asterisks denote injury cavity. (**c**) Human cell marker STEM121 (green) in sagittal section of one animal injected with scNSC neural ribbon segment at 9 days after transplantation. Astrocytes stained with GFAP (red). Viable scNSCs are retained within the cavity and along the cavity wall. Inset is high magnification image of region enclosed by white box wherein scNSC fibers interpenetrate with rat astrocytes. (**d**) High magnification image of cells injected as a suspension depicts cell debris in host spared tissue. (**e**) High magnification image of cells injected in neural ribbons 9 days after grafting. Long STEM121+ processes navigate cavity border. (**f**) High magnification images from (**c**) demonstrates retention of viable cell aggregates in the injury cavity. (**g**) Neural ribbon-delivered scNSCs (green) merge with host parenchyma and co-localize with Synapsin 1 (magenta). Scale bars are 100 µm in (**b**–**f**); 50 µm in (**g**) left panels, 10 µm in rightmost image.

Day 10 scNSC neural ribbons were shipped overnight from Albany, NY to Houston, TX (> 1,750 miles; 20–24 h laboratory-to-laboratory) for in vivo grafting studies in the rat CNS (Fig. 7a). We used a well-characterized and consistent rat model of cervical SCI to assess the ability of neural ribbons to protect and position cervically-patterned scNSCs within the injury site. Two days after shipping, 4-month-old female Long-Evans rats (n = 4) received cervical intraspinal injections of single 3–4 mm neural ribbon segments (NovaCol, 60 µm diameter) containing ~ 5,000 scNSCs (day 12, 1 × 10^8^ cells/ml ribbon concentration) and Fluorescein Dextran 14 days after injury. The number of cells contained within single 3–4 mm segments constitutes an upper limit based on geometric constraints of our extrusion system as well as of the contusion injury size. Two animals received ~ 5,000 cells injected in suspension with vehicle (1,000 cells/µl). Nine days after grafting (injury age day 23, scNSCs day 21), spinal cords were harvested and processed for histological analysis (Fig. 7b–g). Serial longitudinal sections (sagittal plane) revealed that cells injected in suspension outside of neural ribbons were scarcely detected (Fig. 7b) and signal in the spared parenchyma resembled cellular debris (Fig. 7d). However, by injecting single neural ribbon segments, we observed that STEM121 immunopositive viable human scNSCs were retained within the contused injury cavity and began to extend processes (> 500 µm) along the cavity border (Fig. 7c,e,f). scNSCs that merged with host tissue at the cavity border also co-localized with Synapsin 1 nine days after grafting (Fig. 7g). These qualitative data demonstrate a neuroprotective function of the hydrogel platform, supporting the use of neural ribbons to position and retain cells in the SCI cavity even at low delivery doses.

## Discussion

**Figure 8:**
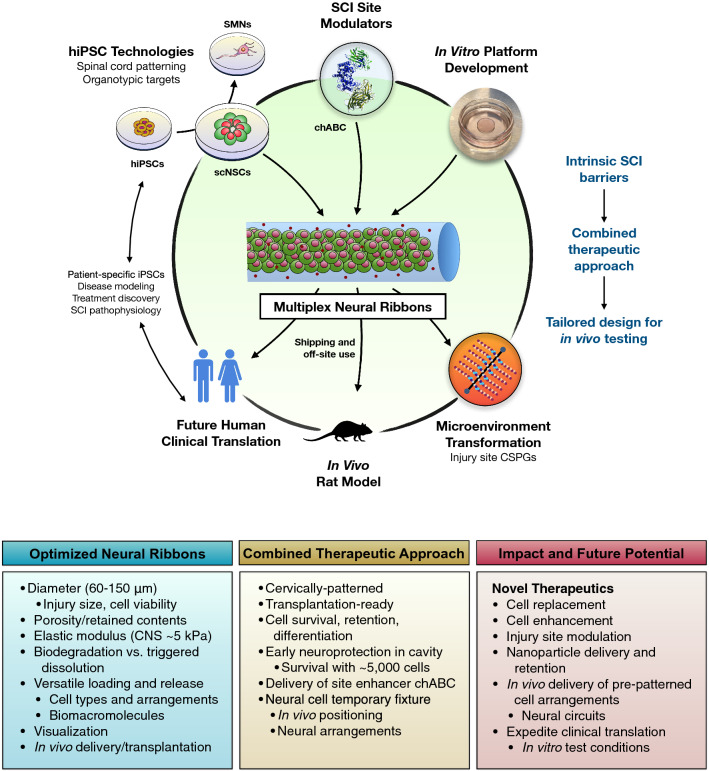
Overcoming barriers to SCI cell therapy with multiplex homotypic neural ribbons. Overview of multifunctional neural ribbons as a novel combined approach to SCI therapy. The hiPSC cell resource can be merged with injury site modulator chABC in hydrogel platforms optimized in vitro. Applications tested here through to animal studies in rat impact technology transfer for future human health and interventions.

Neural ribbons containing homotypic, cervically-patterned NSCs constitute a protective multifunctional platform for cell delivery, retention, and positioning in SCI (Fig. 8). We apply advanced NSC differentiation protocols that focus on spinal cord identity, and along with the neural ribbons, result in enhanced cell survival and retention at the injury site. Several studies in rat, mini-pigs and non-human primates demonstrate the promise of NSC therapeutics but recognize challenges posed by the injury setting^33–39^. An ongoing human clinical trial with isolated human spinal cord-derived NSC line NSI-566 supports clinical safety^40^. However, in these studies, high-dose NSC integration into the injured spinal cord without a delivery platform shows mixed ability to reestablish lost locomotor modalities^35,40^. Contusion injury is the most prevalent form of SCI in humans. Here using a relevant rat C4 hemi-contusion model^16^, we demonstrate that minimally invasive delivery of scNSC neural ribbons by syringe injection into the contused injury cavity reproducibly achieves cell survival and retention with as few as 5,000 cells. Traditional NSC methods in rat models typically deliver cells in a dose range of 200,000 to 2 million cells. Here, the transplanted scNSCs survive and project long processes that co-localize with Synapsin with greater efficiency compared to scNSCs injected outside of the ribbons. While higher cell delivery numbers may be required to fill the cavity of larger injuries, the survival of our small grafts is a proof-of-principle for the neuroprotective function of this platform. Building on foundational work in human stem cell models,^17,18^ Kumamaru et al.^5^ developed a method to stabilize NSCs over several passages while retaining spinal cord identity. We use this early NMp induction method to induce *Hox* gene collinear activation during the first 5 days of differentiation, but converted to neuroectoderm by next modulating RA and Shh signaling pathways to generate scNSCs with cervical regional identity. The approaches described in this work offer strategies that apply to a variety of CNS pathological conditions requiring cell delivery to specified anatomic compartments.

Extrusion-based techniques with alginate and disease-specific requirements for cell types are being explored to develop a higher level of control over therapeutic cells, including incorporation of factors to direct or drive differentiation^29,41^, and ability to engineer structures with increasing complexity^42^, and have seen success in animal models of disease, particularly of diabetes mellitus^42,43^. Here we advance alginate microfiber technology in the form of multiplex neural ribbons applied for the first time to in vitro and in vivo models of SCI. scNSCs were encapsulated as high-density single cells or pre-established neural aggregates at day 10 according to neural rosette cell number and size parameters. scNSCs retained metabolic activity, cell cycling, viability, and multipotent differentiation potential. These parameters were improved by increasing encapsulated cell density tenfold, and further by the loading of scNSC aggregates with pre-established 3-D cell–cell connections versus single cells. It was previously shown that cell–cell interactions and survival are bolstered by including natural ECM components^42^ or chemical modifications such as conjugation with the integrin-binding domain peptide RGD that together more fully recapitulate a natural microenvironment. We therefore compared neural ribbons formed with alginate alone (ProNova) to an alginate-type 1 collagen IPN (NovaCol) and RGD-conjugated alginate with type 1 collagen IPN (RGD-Col) (Fig. 4; see also Table S3). scNSC viability was highest using the RGD-Col composition, and encapsulated cell–cell connectivity was promoted by smaller 60 µm ribbon diameter versus 100 µm or 150 µm diameters. Since the RGD modification may exacerbate potential fibrotic responses of host cells in vivo, 60 µm diameter NovaCol neural ribbons were chosen for in vivo grafting studies. Importantly, we show the ability of scNSCs to form robust calcium-dependent adherens junctions within NovaCol neural ribbons, and to simultaneously differentiate and penetrate into surrounding 3-D ECM in vitro on a time scale similar to alginate biodegradation.

We demonstrate neural ribbon multifunctional customization by co-encapsulating chABC in vitro. However, chABC was excluded in our in vivo pilot study due to ongoing debate of the role of the glial scar in regeneration and repair. Our data support the premise that younger NSCs do not require chABC for initial navigation into host tissue, but acknowledge that chABC may be required for transplanting specialized postmitotic neurons and that this need may vary between subjects.

The ability to ship long-distance intact and viable neural constructs that retain stemness has not been previously demonstrated in the stem cell community and further highlights the broad clinical applicability of this approach to facilitate future GMP SCI therapeutic schemes. The ability to overcome multiple central barriers to SCI cell therapy remains a challenge, but is expected to be achievable by advancing knowledge of the appropriate cell type and reproducible delivery method.

## Methods

### Human hiPSC culture and maintenance

The self-designated African American human iPSC line F3.5.2 was previously developed by the Paluh and Cibelli labs using lentivirus transduction of donor fibroblasts with Yamanaka factors and extensively characterized^19,20^. Here, F3.5.2 was maintained in mTeSR1 or mTeSR Plus complete growth medium (STEMCELL Technologies) with 1 × penicillin–streptomycin (P-S; Gibco) on hESC-qualified Corning Matrigel substrate (37 °C, 5% CO_2_). hiPSCs were passaged every 4–7 days as 50–100 µm cell aggregates using the enzyme-free Gentle Cell Dissociation Reagent (GCDR, STEMCELL Technologies) 1:6 in 6-well plates. Cells were stored using serum-free mFreSR cryopreservation medium (STEMCELL Technologies) at passage number P14–P32. G-band karyotyping of hiPSCs was performed to validate genomic integrity (Cell Line Genetics, Madison, WI).

### hiPSC-derived NMps and scNSC rosettes

F3.5.2 hiPSCs were grown as adherent colonies on Matrigel-coated cultureware to ~ 60% confluency. On day 0 of differentiation, colonies were rinsed 2 × in DMEM/F-12 prior to addition of fresh Stage 1 Neuromesodermal Progenitor Medium (NMPM). N2B27 basal medium: 1:1 DMEM/F-12:Neurobasal Plus medium, 2% (vol/vol) B-27 Plus supplement, 1% (vol/vol) N-2 supplement, 1 × GlutaMAX, 1 × MEM Non-Essential Amino Acids, 1 × P-S (Gibco); NMPM supplements: 40 ng/ml recombinant human (rh) FGF2 (RnD), 40 ng/ml rhFGF8 (RnD), 2 µM CHIR99021 (Tocris), 10 µM DAPT (Sigma), 10 µM SB431542 (Tocris), 100 nM LDN193189 (Tocris), 0.36 U/ml heparin (Sigma). NMPM was changed daily to day 5, at which point NMps were rinsed and passaged as small aggregates using GCDR (1:2 high-density, 6-well plates) in Stage 2 Cervical Patterning Medium (CPM). CPM: N2B27 supplemented with 100 nM Retinoic Acid (Sigma), 200 nM Hh-Ag1.5. 10 µM Y-27632 (ROCK inhibitor; Tocris) was used only during passages and fresh medium was replaced the following day. In Stage 2 CPM, early scNSC rosettes were visible by day 6–7 and grew in size to day 10. scNSC cultures were passaged 1:3 on day 10 from 6-well to 12-well plates, grown to confluency and maintained for 2–3 days prior to additional high-density passaging (1:2). Cells were cultured on hESC-qualified Matrigel substrate throughout the entirety of differentiation. For culture purification, MNP/SMN differentiation, and axon outgrowth, short-term 24 h neurospheres were formed in ultra-low attachment 6-well plates (Corning) using a Scilogex MX-M microplate mixer (120 rpm) and plated onto freshly coated substrates.

### Differentiation to MNPs and SMNs

Cervically-patterned scNSCs were maintained in Stage 2 CPM from day 5 to 25. At day 25, CPM was changed to Terminal Differentiation Medium (TDM) to promote maturation of post-mitotic SMNs. TDM: N2B27 supplemented with 10 ng/ml rhBDNF (RnD), 10 ng/ml rhGDNF (RnD), 1 µM dibutyryl cyclic-AMP (Sigma), 20 µg/ml ascorbic acid (Sigma). ½ volume of conditioned medium was replaced with fresh medium every 3–4 days.

### Phase contrast, fluorescence microscopy and immunofluorescence

Phase contrast images were acquired using two systems: (1) Nikon Eclipse TS100 microscope equipped with 4×/0.10, 10×/0.25 Ph1 ADL and 20×/0.40 Ph1 ADL phase contrast objectives with Qimaging Retiga 2000R camera and NIS Elements-D acquisition software; (2) Zeiss Invertoskop 40C microscope equipped with 5×/0.12 CP-Apochromat, 10×/0.25 Ph1 A-Plan and 20×/0.30 Ph1 LD A-Plan objectives mounted with Zeiss Axiocam 202 mono camera. For immunofluorescence (IF) characterization of hiPSCs and differentiation protocol efficiency, cells were plated in Matrigel-coated glass-bottom imaging chambers (Invitrogen). Lab-Tek II 4-chambered cover glass (Nunc, #155382) was primarily used. Cells were allowed to adhere 1 to 2 days prior to fixation or were differentiated directly for analysis at specified time points. For sample preparation, culture medium was aspirated and cells were rinsed once in 1 × PBS (37 °C). Cells were then fixed at 37 °C with filtered 10% buffered formalin for 30 min. Samples were rinsed three times in 1 × PBS and permeabilized for five min in 0.1% Triton X-100. Fixed and permeabilized samples were rinsed two more times with 1 × PBS and blocked for 30 min in 1% BSA fraction V. Primary antibodies were then added for overnight incubation at 4 °C rocking or room temperature (Supplementary Table 1 primary antibodies). The next day, samples were rinsed and incubated with DAPI and the appropriate species- and immunoglobulin-matched AlexaFluor secondary antibodies (Invitrogen) for 1 h in the dark at 4 °C. Following three additional rinse steps, cells were imaged directly in chambered cover glass wells. Fluorescence microscopy was performed primarily using Zeiss Axio Observer.Z1 inverted fluorescence microscope equipped with 20×/0.8 air and 63×/1.4 oil Plan-Apochromat DIC objectives. An Hamamatsu ORCA ER CCD camera and the Zeiss AxiovisionRel software (ver. 4.8.2) were used for image acquisition. For fluorescence microscopy of adherent cells, 6- to 20-slice Z-stacks were gathered at 1 µm separation distance and compressed using the Extended Focus feature in post-acquisition processing. For identical antibodies, uniform exposure times were maintained across samples. Images were compiled in Keynote and, if necessary, adjusted linearly for brightness. For images requiring 10 × magnification, a Leica confocal TCS SP5 II system was used with the 10×/0.30 HCX PL FLUOTAR air objective lens. Leica Application Suite Advanced Fluorescence software was used for image acquisition.

### In vitro CSPG glial scar models and chABC activity

We modeled aspects of the glial scar inhibitory microenvironment in SCI using the well-established Laminin-CSPG gradient spot assay^31,32^. Briefly, 2 µl CSPG spots (0.4 mg/ml Aggrecan, 10 µg/ml Laminin in HBSS) were plated onto PDL-Nitrocellulose coated chambers of Lab-Tek II cover glass (Nunc #155382; 2–5 CSPG spots/chamber) and allowed to air dry. Dried spots were stable in HBSS or cell culture media for > 1 week at 4 °C. To visualize the inverse CSPG gradient, spots on coverslips were incubated overnight in 1% BSA (1 × PBS) with the anti-CS-56 monoclonal antibody against CSPG (1:1,000), rinsed 3X in HBSS and incubated 1:1,000 with donkey anti-mouse AlexaFluor 594 secondary antibody for microscopy. We grew day 10 scNSCs as well as differentiating neuronal cultures on CSPG spots coated with fresh Matrigel to test the efficacy of the CSPG gradient to exclude neural cells. The outer rim of CSPG spots was sufficient to exclude both scNSCs and neuronal axon. CSPG spots were incubated with 0.1 U/ml purified chABC in aCSF for 3 h and analyzed for enzymatic digestion (CS-56 IF) to test chABC activity. Relative fluorescence intensities of treated spots compared to controls were measured in ImageJ using nearly identical fields of view. aCSF and cell culture media both provide Ca^2+^ cation that is a necessary cofactor for chABC enzymatic activity.

chABC activity was also tested in multiple assays exploiting the endogenous production of CSPG ECM by scNSCs. To determine the time to achieve full chABC effect, day 10 scNSC rosettes were seeded into chambered cover glass at high density and grown to confluency. chABC was then added at 0.1 U/ml in culture medium and returned to 37 °C. Cells were fixed at 30 min intervals to 120 min and compared to controls (no chABC treatment). CS-56 fluorescence intensity (a.u.) was quantified over time (mean ± s.e.m.). n = 7 fields were quantified for each time point (N = 35 total measurements). scNSCs were seeded identically for chABC addition and washout studies. First, cells were fixed at three-hour intervals to determine CS-56 baseline fluorescence intensity. At 6 h, 0.1 U/ml chABC was added and samples were fixed at three-hour intervals to 12 h. The remaining samples were then washed 4 × in cell culture medium to dilute chABC and again fixed at three-hour intervals to 18 h, at which point samples were fixed at six-hour intervals to up to 36 h. n = 4 separate fields were measure per time point (N = 40 total measurements). We combined Ki-67 IF with the dual apoptosis kit described previously to quantify the effect of chABC on scNSC proliferation and apoptosis. Cells were fixed at t = 24 and 48 h after addition of 0.1 U/ml in culture medium. The percent of nuclei expressing Ki-67 versus dual apoptotic Casp3S-AnnV markers were quantified and compared to no chABC treatment controls (n = 4 fields averaged per condition). Vybrant MTT (ThermoFisher) chromogenic assays were also used to measure effect of chABC on cell proliferation. Acellular culture medium alone was used as a negative control. scNSCs were seeded into Matrigel-coated 96-well plates and grown to ~ 60% confluency, at which point 50% of wells containing scNSCs received 0.1 U/ml chABC. MTT measurements were performed at t = 24 and 48 h after chABC addition according to manufacturer’s instructions. Briefly, 10 µl of 12 mM MTT stock was added and incubated in the dark at 37 °C for 3 h. Insoluble formazan was then dissolved in SDS-HCl at 37 °C and absorbance was measured at 570 nm using an automated plate-reader. The experiment was performed in triplicate and averaged.

### Neural ribbon scNSC and chABC encapsulation

Ultrapure sodium alginates tested in this study are summarized in Supplementary Table 4 (Dupont Novamatrix). Lyophilized sodium alginate was resuspended in 0.9% sterile normal saline to 1.5% (w/w) and stored in solution at 4 °C as aliquots. For extrusion of cell-free ribbons, we used a sterile 3 ml BD syringe with stainless steel precision dispensing tips that attach by luer lock. Three needle tip sizes were 60 µm (34G), 100 µm (32G) and 150 µm (30G) inner diameter (Nordson EFD). 35 to 75 µl of 1.5% sodium alginate was pipetted directly into the luer lock tip, avoiding bubbles, and dispensed into 100 mM sterile CaCl_2_ within individual wells of a multi-well plate (12-, 24-, 48-, or 96-wells; assay-dependent), or pipetted directly onto a sterile glass microscope slide or glass bottom imaging chambers (Invitrogen). For multi-well plates, the BD syringe and Nordson tip were oriented orthogonally to the CaCl_2_ liquid surface. Initial pressure was applied to the extruded syringe plunger and then immediately submerged into CaCl_2_ solution while maintaining continuous pressure. This method is useful to form both single ribbons using the entire loaded volume as well as multiple ribbons in individual wells by withdrawing the Nordson tip and re-submerging promptly into a fresh volume of CaCl_2_. Ribbon quality was monitored by visual inspection using phase contrast microscopy. To generate NovaCol and RGD-Col IPN ribbons, non-gelled PureCol EZ gel solution (5 mg/ml; Advanced Biomatrix, #5074) was combined 1:3 with 1.5% alginate (ProNova or Novatach LVM GRGDSP, respectively) and mixed thoroughly prior to extrusion. To prevent early gelation of collagen, reagents were kept at room temperature. However, subsequent incubation at 37 °C for 30–45 min drives gelation of collagen to establish the IPN. Ribbon compositions used in this study are summarized in Supplementary Table 3.

To encapsulate scNSC single cell suspensions, rosettes were dissociated using Accutase or TrypLE for 5 min at 37 °C, distributed to 1.5 ml Eppendorf tubes 1:1 with DMEM/F-12 and counted. scNSCs were centrifuged at 350×*g* for 5 min followed by complete aspiration of supernatants. The scNSC pellet was resuspended in alginate or alginate-collagen mixtures to concentrations of 1 × 10^7^ or 1 × 10^8^ cells/ml by gentle static mixing with the pipette tip, avoiding bubbles. The scNSC-hydrogel suspension was then loaded directly into the Nordson tip and extruded into CaCl_2_ using a syringe as described above. CaCl_2_ was aspirated immediately after crosslinking with a 1,000 µl pipet tip. Ribbons were suspended in culture medium, inspected, and returned to the incubator. To load scNSC aggregates (50–100 µm), GCDR with mechanical passaging was used in place of Accutase/TrypLE dissociation. The same number of wells at 100% confluency (ave. 3.5 × 10^6^ cells/well in 12-well plate) were used to generate neural aggregate ribbons as for the 1 × 10^8^ cells/ml condition. For chABC encapsulation, purified enzyme was added to alginate suspensions prior to extrusion for a final working concentration of 0.1 U/ml.

### scNSC dose, proliferation, and viability in neural ribbons

Cell dose (i.e. cell number per unit ribbon length) was determined experimentally at SUNY Polytechnic Institute and compared to values obtained by Houston Methodist after shipping. scNSC neural ribbons were formed directly on glass slides, rinsed in deionized water and manually severed into to 3 mm segments. Ribbon segments were individually dissolved in 20 µl 1.6% sodium citrate (Ca^2+^ chelation) and counted both by hemocytometer and automated cell counting. Only ribbons formed using 1 × 10^8^ cell/ml single cell suspension or corresponding neural aggregates (50–100 µm) were subject to this analysis. For neural aggregates, only segments containing cells along the entire 5 mm length were used. After alginate dissolution, aggregates were dissociated completely prior to counting. n = 5 neural ribbon segments were counted for each loading configuration (N = 10 segments total).

Cell metabolic activity was assessed using CellTracker dye 24 h post-encapsulation. The Vybrant MTT kit (ThermoFisher) was additionally used according to manufacturer’s instructions and insoluble Formazan crystals in ribbons were imaged by phase contrast. For cell proliferation within ribbons, we combined Ki-67 IF with CellTracker and DAPI counterstain and acquired images of mitotic figures using high-magnification fluorescence microscopy (Supplementary Tables 1, 2). We investigated scNSC apoptosis after CaCl_2_ incubation for 1, 2 and 5 min versus control (no CaCl_2_) to test the effect of 100 mM CaCl_2_ on scNSC viability over a timespan relevant to ribbon formation. Culture medium was aspirated from adherent scNSCs seeded in chambered cover glass (Nunc #150680) and rinsed once in warm 1 × PBS prior to addition of sterile 100 mM CaCl_2_. At an appropriate timepoint, CaCl_2_ was aspirated and cells were rinsed twice with 1 × PBS and returned to the incubator in prewarmed cell culture medium for 2 h. Dual apoptosis markers that are a novel 488 NucView Caspase-3 substrate and TexasRed (TR)-AnnexinV (Biotium #30067) were used for fluorescence imaging and quantification according to manufacturer’s instructions. As a positive control, apoptosis of scNSCs in ribbons was induced by addition of 1 mM H_2_O_2_ in cell culture media 24 h prior to staining. scNSCs with dual staining for NucView 488 Caspase-3 substrate and TR-AnnexinV were counted manually in ImageJ. For each timepoint, scNSC neural ribbons incubated in parallel were dissolved by 1.6% sodium citrate and re-plated on freshly-coated Matrigel multi-well plates. For Trypan blue exclusion assays, we formed a 40 µl ribbon at 1 × 10^8^ cells/ml and incubated in CPM for 24 h. Following alginate dissolution in 1.6% sodium citrate, cells were dissociated by gentle pipetting, stained with Trypan blue dye, and viability determined by hemocytometer.

### Neural ribbon-encapsulated chABC in vitro assays

Using similar in vitro glial scar and CSPG ECM assays as described in the previous section, we tested the ability of neural ribbon-encapsulated chABC to ameliorate the inhibitory CSPG microenvironment. For these experiments, we formed single 40 µl ribbons containing enough chABC to establish a working concentration of 0.1 U/ml in 0.5 ml of medium, assuming 100% release. As an initial test, single ribbons were incubated with CSPG spots in aCSF and fixed for CS-56 fluorescence at 24 h. For cellular assays, day 10 scNSCs were seeded onto Matrigel-coated CSPG spots (1,500 cells/mm^2^) and grown to confluency. scNSCs were then incubated with empty (negative control) or chABC-containing ribbons in 0.5 ml CPM for 24 h. scNSC crossing events per CSPG spot were quantified by manual counting. The same assay was repeated with differentiating neuronal cultures (1,500 cells/mm^2^) or plated neurospheres grown in suspension (5–8 neurospheres/well). For neuronal cultures, the experiment was initiated when sufficient axon outgrowth was observed by phase contrast 3–4 days after seeding. n = 9 full CSPG spots quantified per condition for scNSCs and neurons (N = 36 total spots). For microscopy, ribbons were removed from chambers prior to fixation and ICC with 1 × PBS-based solutions. We embedded neural ribbons in scNSCs and Type 1 collagen gel. scNSCs were grown to 100% confluency on Matrigel-coated glass bottom dishes (12 mm viewing area; Nunc, #150680). TexasRed-Dextran ribbons were formed +/− chABC, rinsed and manually positioned onto scNSCs. The scNSC/NR combined culture was immediately embedded in 2.5 mg/ml PureCol EZ gel and incubated overnight at 24 h (0.5 ml culture medium), at which point, cultures were co-stained for DAPI/CS-56 and quantified for CS-56 immunoreactivity (fluorescence intensity; n = 9 fields for no chABC, n = 7 fields for +chABC). For the latter assay, ICC was performed in the PIPES/HEPES/CaCl_2_ buffer to preserve neural ribbon integrity.

### Neural ribbon long-distance shipping and receiving

The Houston Methodist Neuroregeneration lab is > 1,750 miles from SUNY Polytechnic CNSE in Albany, NY. For long-distance shipping of scNSC neural ribbons encapsulated at day 10 (FedEx overnight, ~ 20–24 h laboratory-to-laboratory), we used a battery-powered portable incubator with removable block (CryoLogic BioTherm INC-12V). Prior to shipping, scNSC neural ribbons were formed directly in 12-well plates and resuspended in CPM supplemented with 10 µM ROCK inhibitor. Ribbon suspensions were then transferred to 1.8 ml cryovials using wide bore 1,000-µl pipette tips. Neural ribbon transport stability depends both on physical and chemical factors, including cell–cell contacts that are promoted by high densities, and prevention of necrosis due to reduced diffusion of gases and nutrients.^30^ Upon receipt, neural ribbons were resuspended in fresh medium. Cell recovery after shipping was tested by dissolving alginate in 1.6% sodium citrate and plating of recovered cells onto freshly-coated Matrigel cultureware. In parallel, a group of neural ribbons were kept intact for in vivo injection in rodent C4 hemi-contusion injury models.

### Rat cervical spinal cord hemi-contusion injuries and grafting

Lateral right-sided C4 hemi-contusion lesions were induced with moderate displacement (0.8 mm) in 4-month-old female Long-Evans rats (N = 6) as previously described^16^. Four animals received neural ribbons and two animals received scNSCs delivered in suspension. Briefly, the C4 laminae was exposed for right-sided hemi-laminectomy in anesthetized animals. The animal was then placed in a custom spinal frame, clamping the lateral processes of C3 and C5. An electromagnetic probe (Ling Inc.) was positioned at the surface of the dura with an initial sensing force followed by rapid 0.8 mm displacement with a dwell of 14 ms. Afterward, muscles were sutured in layers and the skin was clipped. Contused animals were immunosuppressed with cyclosporine-A (CSA; 10 mg/kg, *i.p.*) two days prior to the human neural ribbon transplantation. The CSA regimen was maintained until euthanasia. For grafting studies, the contused spinal cord was re-exposed 14 days after injury in anesthetized animals and the lateral process of C3 was clamped. 60 µm diameter NovaCol ribbons containing human scNSC aggregates and Fluorescein Dextran were manually severed into 3–4 mm segment lengths. Single segments containing approximately 5,000 cells were drawn into a 26G curved needle Hamilton syringe with a 45° bend ~ 2 mm proximal to the bevel. An Hamilton syringe was placed in a Kopf micromanipulator fixed to the spinal frame and positioned 0.5 mm lateral to the midline ipsilaterally. Dura was punctured by the syringe tip and advanced to 0.7 mm depth. A single ribbon segment was injected over 1 min in 5 µl of HBSS vehicle per animal. The needle was maintained in position for 2 min dwell, followed by 100 µm retraction and another 2 min dwell. The needle was slowly retracted while monitoring for efflux of fluid or the ribbon segment onto the surface of the dura. Finally, muscle and skin layers were sutured and the animals received standard post-operative care. All rat experimental protocols were approved by Houston Methodist Research Institute IACUC and carried out in accordance with relevant ethical guidelines and regulations. This includes animal comfort, veterinary care, methods and reasons for euthanasia, and materials and hiPSC-derived neural injections.

#### Tissue processing and immunohistochemistry

Nine days after neural ribbon injection, rats were sedated with isoflurane and transcardially perfused first with cold 0.1 M PBS with 10,000 U of heparin followed by 4% paraformaldehyde in 0.1 M PBS (pH 7.4). The spinal cord was harvested and stored in 4% paraformaldehyde at 4 °C overnight. For cryoprotection, the spinal cords were passed through a sucrose buffer gradient (10%, 20%, 30%; 24 h per solution) at 4 °C. Cords were then aligned longitudinally in a Tissue Tek OCT block (Sakura, Nederland) using dry ice, and stored at − 80 °C. 20 µm thick sagittal sections were prepared using a Cryostar NX50 cryomicrotome (ThermoFisher). Serial sections of the entire spinal cord were then mounted onto positively-charged slides (Fisher Scientific). For immunohistochemistry, slides were first blocked with 10% goat or donkey serum in 0.2% Triton X-100-PBS (T-PBS) for 1 h at room temperature followed by primary antibody incubation in 1% serum, 0.2% T-PBS overnight at 4 °C. Primary antibodies used were mouse anti-STEM121 (1:500; Takara), chicken anti-GFAP (1:3,000; Abcam), rabbit anti-IBA1 (1:1,000; Wako), rabbit anti-Synapsin 1 (1:200; Abcam). Primary antibody staining was performed on the same day across groups. After three additional wash steps with 1% serum T-PBS (1–2 min/wash), slides were incubated with secondary antibodies in PBS for 1 h. Secondary antibodies used were donkey anti-mouse AF647 (1:800; ThermoFisher), donkey anti-chicken Rhodamine (1:200; Jackson Immunoresearch), donkey anti-rabbit 488 (1:800; Jackson Immunoresearch), donkey anti-rabbit Cy5 AffiniPure (1:400; Jackson Immunoresearch). Slides were mounted with DAPI-Permount (Fisher Scientific) and imaged by confocal microscopy.

#### Statistical analysis and reproducibility

Raw data were compiled in Microsoft Excel (v16.16.16) and exported to GraphPad Prism (v8.3.0) for statistical analysis and plot-generation. All data is presented as (mean ± s.e.m.) and analyzed using unpaired two-tailed t-test unless otherwise specified in figure legends. ImageJ (NIH) was used for data analysis of images. Cells were counted manually for quantification of IF data as average percentages. One-way ANOVA test was used to compare ribbon diameters across groups and relative fluorescence intensity of CSPG spots. ****p < 0.0001, ***p < 0.001, **p < 0.01, *P < 0.05, n.s. not significant (α = 0.5). Detailed information for each experiment is provided in the figure legends.

Figures were compiled in Keynote (v9.2.1). Schematics were drawn in Canvas Draw (v4.0.1) or Keynote. High resolution pdfs of small file size were generated from EPS files using the CellPress plugin (cellpressArtwork.joboptions) with Adobe Acrobat. Plots were generated in GraphPad Prism (v8.3.0) and modified in Keynote.

## Supplementary information

Supplementary Information 1.

Supplementary Information 2.

Supplementary Information 3.
