# Supplementary material for: Fabrication of homotypic neural ribbons as a multiplex platform optimized for spinal cord delivery
Source: Sci Rep. 2020 Jul 31;10:12939. doi: 10.1038/s41598-020-69274-7 (PMC7395100; doi:10.1038/s41598-020-69274-7)
Supplement: Supplementary file 1 — Supplementary Information 1. [file 41598_2020_69274_MOESM1_ESM.docx]

**Supporting Information**

**Fabrication of homotypic neural ribbons as a multiplex platform optimized for spinal cord delivery**

Zachary T. Olmsted, Cinzia Stigliano, Abinaya Badri, Fuming Zhang, Asher Williams, Mattheos A. G. Koffas, Yubing Xie, Robert Linhardt, Jose Cibelli, Philip Horner and Janet L. Paluh^*^

**Corresponding author:** Dr. Janet Paluh

State University of New York Polytechnic Institute CNSE

4424 NanoFab East

257 Fuller Road, Albany, NY 12203

Phone: (518) 956-7047

Fax: (518) 437-8687

Email: jpaluh@sunypoly.edu

**Supplemental Figures**

**Supplementary Figure 1. Hydrogel ribbon retention of biomacromolecules, biodegradation, and shipping. a,** (**i**) Top: empty ribbons demonstrate low background autofluorescence using Fluo-4 AM calcium indicator. Dotted white lines outline ribbon edge. Bottom: ribbon visualization by loading with Fluorescein-DHPE phospholipid. (**ii**) IF visualization of ribbons co-packaged with AlexaFluor secondary antibodies donkey anti-goat 594 (DG594; magenta) and goat anti-mouse 488 (GM488; cyan) at 1 h (top) and 24 h (bottom) after ribbon formation. Select antibody puncta are circled. (**iii**) Retention of high MWT fluorescent Dextran conjugates co-extruded in 1.5% alginate ribbons at 24 h. Top: 70 kDa TexasRed-Dextran conjugate. Bottom: 500 kDa Fluorescein-Dextran conjugate. **b**, Bulk alginate hydrogel biodegradation using aCSF. Percent gel remaining by wet weight of bulk alginate is plotted. n = 3 bulk measurements per timepoint. Recovered ribbons incubated in aCSF from corresponding days 1 (intact) and 21 (degraded) are shown immediately following glass coverslip application. n = 3 ribbons per time point. **c**, White light photos of scNSC-laden neural ribbon step-wise handling. Yellow arrows point to suspended cell-laden neural ribbon visualized during handling. Scale bars are 50 µm.

**Supplementary Figure 2. Purified chABC modulates the endogenous scNSC CSPG microenvironment. a**, scNSC rosette cultures produce abundant endogenous CSPG ECM. Immunostaining with CS-56 antibody in red. Cells are counterstained with F-Actin and DAPI (nuclei). **b**, High-magnification image of single scNSC rosette (day 10). F-Actin/CS-56/DAPI. **c**, RNA-Seq normalized counts of known functional inhibitory CSPG receptors at specified days in differentiation from scNSC to SMN. **d,** Incubation with 0.1 U/ml chABC reduces endogenous CSPG signal to the intracellular perinuclear region (white arrow). CS-56/DAPI. **e**, Time-course of chABC effect using scNSCs. Confluent scNSCs were incubated with 0.1 U/ml chABC and fixed at t = 30, 60, 90, 120 min. CS-56 CSPG signal is restricted to the intracellular perinuclear region by ~60 min. **f**, Quantification of IF data in (**e**) by fluorescence intensity (a.u.). Red dot indicates chABC addition. **g**, scNSCs resynthesize CSPG ECM after chABC washout. 0.1 U/ml chABC was added at t = 6 h (6 h incubation) and washed out at t = 12 h. CS-56 CSPG signal is co-stained with F-Actin and DAPI. **h**, Quantification of IF data in (**g**) by fluorescence intensity (a.u.). Red dot indicates chABC addition, blue dot indicates chABC washout. **i**, chABC incubation (0.1 U/ml) does not impair scNSC proliferation or reduce viability. Fixed scNSCs stained with cell cycle biomarker Ki-67 (punctate nuclear localization) and dual apoptosis markers Casp3S/TR-Annexin V. **j**, Quantification of IF data in (**i**) as percent of nuclei. scNSCs were fixed at t = 24, 48 h. n = 4 separate fields were averaged per condition. (one-way ANOVA, n.s.). **k**, MTT assay of scNSCs incubated with 0.1 U/ml chABC for t = 24, 48 h. Cell culture medium with and without scNSCs was used as a positive and negative control, respectively. n = 3 replicates per condition. (two-tailed t test, n.s.). Scale bars are 50 µm.

**Supplemental Tables**

**Supplementary Table 1. Primary antibodies used *in vitro***

| **Target** | **Purpose** | **Compartment** | **Company; Cat#** | **Species/Clone** | **Dilution** |
| --- | --- | --- | --- | --- | --- |
| α-tubulin | MT cytoskeleton | Cytoplasm | ThermoFisher; 62204 | Mouse mAb IgG_1_; DM1A | 1:1,000 |
| OCT4 | Pluripotency; NSC negative control | Nucleus | SCBT; sc-5279 | Mouse mAb IgG_2B_; C-10 | 1:500 |
| SSEA-4 | Pluripotency | Cell surface | STEMCELL; 60062 | Mouse mAb IgG_3K_; MC-813-70 | 1:1,000 |
| SOX2 | Pluripotency; NSCs | Nuclear | RnD; MAB2018 | Mouse mAb IgG_2A_; 245610 | 1:1,000 |
| SOX2 | Pluripotency; NSCs | Nuclear | RnD; AF2018 | Goat pAb IgG | 1:1,000 |
| SOX1 | NSCs | Nuclear | RnD; AF3369 | Goat pAb IgG | 1:500 |
| Nestin | NSCs | Intermediate filament cytoskeleton | RnD; MAB1259 | Mouse mAb IgG_1_; 196908 | 1:1,000 |
| Ki-67 | Active cell proliferation | Nuclear puncta, chromosomes | Abcam; ab15580 | Rabbit pAb IgG | 1:500 |
| Pan-Cadherin | Cell-cell adhesion | Surface protein | Abcam; ab6528 | Mouse mAb IgG_1_; CH-19 | 1:1,000 |
| NCAM-1 | Neural cell adhesion | Surface protein | RnD; AF2408 | Goat pAb IgG | 1:1,000 |
| Vimentin | NSCs, glial cells, microglia | Intermediate filament cytoskeleton | RnD; AF2105 | Goat pAb IgG | 1:1,000 |
| ZO-1 | Apical redistribution to adherens junctions in neural rosettes | Tight junctions | ThermoFisher; 33-9100 | Mo mAb IgG_1_; ZO1-1A12 | 1:1,000 |
| Chondroitin sulfate-56 (CS-56/Aggrecan) | Inhibitory ECM glycosaminoglycan in SCI | Extracellular matrix, intracellular vesicular/perinuclear | Sigma; C8035 | Mouse mAb IgM; CS-56 monoclonal | 1:500 |
| NG2 | CSPG4 proteoglycan | Extracellular matrix | eBioscience; 14-6504-80 | Mouse mAb IgG; 9.2.27 | 1:1,000 |
| OTX2 | Anterior forebrain; spinal cord negative control | Nuclear | RnD; AF1979 | Goat pAb IgG | 1:1,000 |
| CDX-2 | Spinal cord identity (*Hox* gene induction) | Nuclear | RnD; MAB3665 | Mouse mAb IgG_1_; 963809 | 1:1,000 |
| Hox-C6 | Cervical/brachial spinal cord; lateral motor column | Nuclear | ThermoFisher; PA5-41479 | Rabbit pAb IgG | 1:500 |
| Brachyury | Neuromesodermal progenitors (NMps) | Nuclear | RnD; AF2085 | Goat pAb IgG | 1:200 |
| PAX6 | Neuroectoderm | Nuclear | DSHB; PAX6 | Mouse mAb IgG_1_; PAX6 | 1:500 |
| Nkx6-1 | Ventral spinal cord progenitors | Nuclear | DSHB; F55A12 | Mouse mAb; F55A12 | 1:250 |
| OLIG2 | pMN spinal cord motor neuron progenitors; OPCs | Nuclear | RnD; AF2418 | Goat pAb IgG | 1:500 |
| TUJ1 (β-III-tubulin, TUBB3) | Neuron-specific β-tubulin isotype | Neuronal MT cytoskeleton | BioLegend; 802001 | Rabbit pAb IgG; Poly18020 | 1:1,000 |
| SMI312 | Neurofilament-M+H phosphorylated | Pan-axonal cocktail (phospho-NF) | BioLegend; 837904 | Mouse mAb IgG_K_/IgM_K_ cocktail; SMI312 | 1:1,000 |
| NeuN | Post-mitotic neurons | Nuclear | Millipore; MAB377 | Mouse mAb IgG_1_; A60 | 1:1,000 |
| ISL-1&2 | Motor neurons | Nuclear | DSHB; 39.4D5 | Mouse mAb IgG_2B_; 39.4D5 | 1:500 |
| HB9 (MNX1) | Motor neurons | Nuclear, cytoplasmic | DSHB; 81.5C10 | Mouse mAb IgG_1K_; 81.5C10 | 1:100 |
| ChAT | Cholinergic neurons | Soma, neurites, pre-synaptic terminals | RnD; MAB3447 | Mouse mAb IgG_1_; #334008 | 1:1,000 |
| PE-CD271 (NGFR/p75NTR) | Cholinergic neurons | Neuron cell surface | BioLegend; 345106 | Mouse mAb IgG_1K_; ME20.4 | 1:1,000 |
| FOXP1 | Lateral motor column (LMC; appendicular musculature) | Nuclear | Santa Cruz Biotechnologies; sc-398811 | Mouse mAb IgG_1_; A-2 | 1:1,000 |
| Peripherin | Lower motor neurons (PNS) | Intermediate filament cytoskeleton | Santa Cruz Biotechnologies; sc-377093 | Mouse mAb IgG_2A_; A-3 | 1:1,000 |
| Synapsin 1 (SYN1) | Pre-synaptic terminals; NMJ interrogation | Pre-synaptic terminals | Millipore; AB1543 | Rabbit pAb IgG (serum) | 1:1,000 |
| O4 | OPCs | OPC/OL surface sulfatide | Chemicon; MAB345 | Mouse mAb IgM; 81 | 1:500 |
| GFAP | Astrocytes | Intermediate filament cytoskeleton | RnD; MAB2594 | Mouse mAb IgG_1_; #273807 | 1:1,000 |
| PAX7 | Myogenic precursor cells | Nuclear | RnD; MAB1675 | Mouse mAb IgG_1_; #PAX7 | 1:1,000 |
| Desmin | Myogenic precursor cells | Sarcoplasm; intermediate filament cytoskeleton | RnD: AF3844 | Goat pAb IgG | 1:1,000 |
| Myosin Heavy Chain | Striated muscle; NMJ interrogation | Sarcoplasm; actin cytoskeleton | RnD; MAB4470 | Mouse mAb IgG_2B_; #MF20 | 1:1,000 |

**Supplementary Table 2. Fluorescent probes and dyes**

| **Probe** | **Significance** | **Compartment** | **Company; cat#** | **Working concentration or dilution from stock** |
| --- | --- | --- | --- | --- |
| Fluorescein-DHPE | Phospholipid-loaded ribbon | N/A | Invitrogen; F362 | 1:30 in alginate (0.5 mg/ml ethanolic stock) |
| Fluorescein-Dextran 500 kDa (anionic) | Dextran-loaded ribbon | N/A | Invitrogen; D7136 | 1:30 in alginate (10 mg/ml stock) |
| TexasRed-Dextran 70 kDa (neutral) | IgG-loaded ribbon | N/A | Invitrogen; D1864 | 1:30 in alginate (25 mg/ml stock) |
| Hoechst 33342/NucBlue | Nucleic acids | Nucleus | Invitrogen; R37605 | 1 ug/ml |
| Trypan blue | Cell death; excluded by intact membranes | Cytoplasm of compromised cells | Invitrogen; 15250061 | 1:200 from 0.4% stock |
| NucView 488 Caspase-3 substrate/AnnexinV TexasRed | Living cell dual apoptosis assay (caspase-3 activity; phosphatidylserine exposure) | Nucleus (caspase-3 substrate); outer leaflet plasma membrane (AnnexinV) | Biotium; 30067 | 1:250 in 1x AnnexinV binding buffer (each reagent) |
| CellTracker Green CMFDA Dye | Cell-impermeant thiol reactive intracellular products | Cytoplasm of live cells | Invitrogen; C7025 | 1 µM from 1 mM 100x DMSO stock |
| CellTracker Red CMTPX Dye | Cell-impermeant thiol reactive intracellular products | Cytoplasm of live cells | Invitrogen;  C34552 | 1 µM from 1 mM 100x DMSO stock |
| MTT | MTT conversion to formazan by oxidoreductases | Cytoplasm of metabolically active cells | Invitrogen; V13154 | 1:100 from 12 mM PBS stock |
| Phalloidin 488 | F-Actin high-affinity binding | Actin cytoskeleton | Invitrogen; A12379 | 1:1,000 from methanolic stock |
| Phalloidin 594 | F-Actin high-affinity binding | Actin cytoskeleton | Invitrogen; A12381 | 1:1,000 from methanolic stock |
| α-Bungarotoxin 594 | High-affinity nAChR binding | NMJ, cholinergic synapses | Invitrogen; B13423 | 1:1,000 from 1 mg/ml DMSO stock |
| Fluo-4 AM | Cell permeant calcium dye | Cytoplasm | Invitrogen; F14201 | 1:1,000 from 1 mg/ml DMSO stock |

**Supplementary Table 3. Composition of alginate-based neural ribbons**

| **Neural ribbon** | **ProNova UltraPure Alginate**** | **PureCol EZ Gel (Type 1) ^§^** | **Novatach RGD Alginate**** |
| --- | --- | --- | --- |
| ProNova* | + | - | - |
| NovaCol* | + | + | - |
| RGD-Col* | - | + | + |

*60, 100, 150 µm diameters; + component included; - component excluded

**Stock concentration 1.5% dissolved in 0.9% NaCl; final concentration ~1-1.25%

**^§^**Stock concentration 5 mg/ml in DMEM; final concentration ~1.25 mg/ml

**Supplementary Table 4. Properties of alginate used in this study**

| **Alginate** | **MWT (kDa)** | **G/M Ratio** | **Viscosity (mPa*s)** | **Endotoxin (EU/g)** | **Peptide Epitope** | **Solvent** | **%** | **Notes** |
| --- | --- | --- | --- | --- | --- | --- | --- | --- |
| ProNova SLM100* | 150-250 | ≤1 | 170 | <25 | N/A | 0.9% NaCl | 1.5% | Reproducible ribbon formation; chosen for this study |
| ProNova SLG100* | 150-250 | >1 | 204 | <25 | N/A | 0.9% NaCl | 1.5% | Higher mechanical strength; host inflammatory response |
| Novatach LVM GRGDSP (RGD)* | Not given | <1 | Not provided | <10 | GRGDSP (0.015 µmol/mg) | 0.9% NaCl | 1.5% | RGD peptide conjugated (integrin binding domain) |

*Microfiltered 0.22 µm; catalog numbers: 4202301, 4202101, 4270321, respectively

**Supplementary Table 5. Ribbon compatibility in commonly used solutions**

| **Alginate** | **Interpenetrating Network (IPN)** | **Contents** | **Solution** | **Result** | **Time to Dissolve (min)** |
| --- | --- | --- | --- | --- | --- |
| ProNova SLM100 | None | Empty | 154 mM saline | Dissolution | <10 |
|  |  |  | Saline+5 mM glucose | Dissolution | <10 |
|  |  |  | HBSS Ca Mg free | Dissolution | <10 |
|  |  |  | HBSS | Stable | Stable |
|  |  |  | DMEM | Stable | Stable |
| ProNova SLM100 | Collagen | Empty | 154 mM saline | Dissolution | 10-20 |
|  |  |  | Saline+5 mM glucose | Dissolution | 10-20 |
|  |  |  | HBSS Ca Mg free | Dissolution | 10-20 |
|  |  |  | HBSS | Stable | Stable |
|  |  |  | DMEM | Stable | Stable |
| ProNova SLM100 | Collagen | scNSC, chABC | 154 mM saline | Dissolution | 10-20 |
|  |  |  | Saline+5 mM glucose | Dissolution | 10-20 |
|  |  |  | HBSS Ca Mg free | Dissolution | 10-20 |
|  |  |  | HBSS | Stable | Stable |
|  |  |  | DMEM | Stable | Stable |

**Supplementary Table 6. Methods tested for ribbon visualization**

| **Composition** | **Fluorescein-DHPE** | **2^o^ Ab** | **Dextran 70 kDa**** | **Dextran 500 kDa***** |
| --- | --- | --- | --- | --- |
| ProNova* | + | + | + | + |
| NovaCol* | - | - | + | + |
| RGD-Col* | - | - | + | + |

*All diameters; + condition tested, - condition not tested

**Supplementary Table 7. ChABC enzyme source and activity**

| **Genotype** | **Purification System (g+/-)** | **Source** | **Endotoxin** | **Stock**  **Activity (UV-Vis)** | **Working Activity** |
| --- | --- | --- | --- | --- | --- |
| *P. vulgaris* | *E. coli* (g-) | This study | High (>200 EU/unit) | 23.3 U/ml | 0.1 U/ml |
| *P. vulgaris* | *Bacillus* (g+) | This study | None | 6.3 U/ml | 0.1 U/ml |
| *P. vulgaris* | *P. vulgaris* (g-) | AMSBio AMS.E1028-02 | Low (<0.97 EU/unit) | 20 U/ml | 0.1 U/ml |

**Supplementary Table 8. List of strains, plasmids and primers used for chABC expression.**

| **Strain/Plasmid/Primer** | **Description** | **Source/reference** |
| --- | --- | --- |
| **Strains** | | |
| *E. coli* DH5α | General cloning host | Invitrogen |
| *E. coli* BL21*(DE3) | Protein expression host | Invitrogen |
| *E. coli* BL21*(DE3)  pET22b-CSLABC | Chondroitinase ABC producing *E. coli* strain | Shaya et al., 2008 |
| *B. megaterium* MS941 | Defined protease deficient mutant of parent strain (DSM319) *ΔnprM* | MoBiTec GmbH |
| *B. megaterium MS941* pT7-RNAP pPT7-CSLABC | Endotoxin-free chondroitinase producing *B. megaterium* strain | This study |
| **Plasmids** | | |
| pPT7 | T7 promoter, antibiotic resistances against ampicillin (in *E. coli*) and tetracycline (in *B. megaterium*) | MoBiTec GmbH |
| pT7-RNAP | T7 RNAP gene, *xylA* promoter, antibiotic resistances against ampicillin (in *E. coli*) and chloramphenicol (in *B. megaterium*) | MoBiTec GmbH |
| pPT7_CSLABC | pPT7 vector carrying gene coding for His-tagged chondroitinase ABC | This study |
| pET22(b)-CSLABC | T7 promoter, Ampicillin resistance, Chondroitinase ABC gene from *Proteus vulgaris* | Shaya et al., 2008 |
| **Primers** | | |
| NheI_CSLABC_F | 5’-GCTAGCATGGGCAGCAGCCATC-3’ | This study |
| SacI_CSLABC_R | 5’- GAGCTCTCAAGGGAGTGGCGAGAG -3’ | This study |
| pPT7_seq_F | 5’-GGGAGACCACAACGGTTTCC-3’ | This study |
| pPT7_seq_R | 5’-GGTTTGCGCATTCACAGTTCTCC-3’ | This study |

**Supplemental Methods**

**Neuromuscular junctions.** Rat L6 adherent myoblasts (ATCC CRL-1458) were maintained in T25 and T75 tissue culture-treated flasks (DMEM/F-12/10% FBS/1x P-S) and passaged according to ATCC instructions. In short, L6 myoblasts were seeded onto Lab-Tek II 4-chambered cover glass (Nunc #155382) and grown to confluency to promote the formation of multinucleated skeletal myotubes. Neurospheres were seeded onto fused myotubes at ~5-8 per chamber and allowed to extended axons for four days in TDM. At day 4, co-cultures were fixed and prepared for imaging analysis using Synapsin 1 and Myosin-HC antibodies as well as α-bungarotoxin 594 conjugate (**Supplementary Tables 1, 2**).

**chABC purification.**

Culture media, enzymes and reagents

Luria-Bertani (LB) medium was used for overnight cultures and protein expression in *Bacillus megaterium* and *Escherichia coli*. The media were supplemented with the required antibiotics to maintain selective pressure on stably replicating plasmids. Tetracycline and chloramphenicol were used at concentrations of 20 μg/ml and of 40 μg/ml, respectively, for *B. megaterium,* and 50 μg/ml ampicillin was used for *E. coli*. Chemicals and nutrients were obtained from Sigma. Plasmid DNA was prepared by E.Z.N.A plasmid mini kit (OMEGA) and digested DNA fragments were recovered from agarose gel (Bio-Rad) by E.Z.N.A. gel extraction kit (OMEGA). FastDigest restriction endonuclease and Rapid DNA ligation kit were purchased from ThermoFisher.

*E. coli* chABC expression and purification

The plasmid pET22b-CSLABC containing the chABC gene in the pET22b vector was obtained from Cygler and co-workers^44^. *E. coli* BL21*(DE3) cells carrying the above plasmid were grown in LB media containing 50 µg/ml ampicillin overnight at 37°C. A 1 ml aliquot of overnight culture was diluted to 1 l in the same growth media, with the final volume not exceeding 1/5^th^ the maximum flask volume, to facilitate maximum aeration. The flask was incubated at 37°C for 4-5 h at 300 rpm until OD_600_ reaches 0.6-0.8. Temperature was decreased to 22°C and maintained for at least 30 min before adding IPTG to a final concentration of 0.2 mM. The cell culture continued shaking at 22°C overnight (usually 18 h of incubation after induction gives the optimum yield). The cells were harvested by spinning at 6000 × *g* for 15 min, then resuspended in 25 ml of Buffer A (25 mM Tris pH 7.5, 500 mM NaCl and 30 mM Imidazole). The resulting single cell suspension was essential for obtaining the maximum yield. The suspension was transferred to a 50 ml-centrifuge tube and kept on ice during sonication under the following conditions: 3 × 30 s with 8-9 output power and 50% cycle. The lysate was centrifuged at 10,000 × *g* for 30 min then the supernatant was collected and filtered through a 0.45 µm filter. The lysate was loaded on a 10 ml Ni-agarose column which is equilibrated with buffer A at a flow rate of 3 ml/min. The column was washed with 100 ml of buffer A and eluted with a linear gradient of imidazole from 30 mM to 250 mM in buffer A in ~60 min. The activity of the eluent and the purity of the protein was measured by SDS-PAGE, with the target protein having an expected molecular weight of about 120 KDa. A successful preparation yields ~40 mg of chABC from 1 L of bacteria culture.

*B. megaterium* plasmid construction

Commercial pPT7 and pT7-RNAP shuttle vectors were used for protein expression in *B. megaterium* (MoBiTec GmbH). pT7-RNAP vector expresses the T7 RNA polymerase under the xylose inducible P_XylA_ promoter. pPT7 plasmid contains the T7-RNAP promoter that is recognized by T7 RNA polymerase. The chondroitinase ABC gene with an N-terminal histidine tag was amplified by a polymerase chain reaction (PCR) using Accuzyme mix (BIOLINE) from the pET22b-CSLABC plasmid published by Cygler and co-workers^44^. NheI and SacI sites were added to the primers and hence incorporated in the PCR product. Both the amplicon and pPT7 plasmid were digested with restriction enzymes and ligated at the NheI and SacI sites. This plasmid was transformed into *E. coli* DH5α by heat shock of chemically competent cells. Several colonies were selected for colony PCR and the correct construct was verified using double endonuclease digestion followed by DNA sequencing (Genewiz Inc.). The verified construct was then transformed into *B. megaterium* MS941 together with the pT7-RNAP plasmid by protoplast transformation. Colonies from successful transformations were screened for antibiotic resistance and maintained as glycerol stocks. **Supplementary Table 7** summarizes chABC enzymes tested in this study. The primers, plasmids, and strains used are listed in **Supplementary Table 8**.

*B. megaterium* chABC expression and purification

A colony of the engineered *B. megaterium* strain co-transformed with the target constructs was picked from a streaked 20 μg/ml chloramphenicol and 40 μg/ml tetracycline agar plate to inoculate 5 ml of LB media in a 15 ml round-bottom culture tube. It was left overnight shaking at 37˚C and 225 rpm, inclined at an angle of ~55˚, for no longer than 12 h. A 0.5 ml aliquot of this seed culture was added to 50 ml of LB medium, in a 250 ml Erlenmeyer flask so that the initial optical density at 600 nm (OD_600_) was ~0.05. The cell culture was incubated in a rotary air shaker (New Brunswick Scientific Innova 44R) at 37 °C, 225 rpm. When OD_600_ was measured to be ~0.250 (after about 4 h of growth), protein expression was induced by addition of (D)-xylose at a final concentration of 0.5% (w/v). The protein expression was continued at 37 °C, 225 rpm overnight (20 h).

The cells were then separated from the growth medium by centrifugation at 4°C (5,500 × *g* for 10 min). The cell pellet was lysed, and soluble lysates were isolated using CellLytic B+ Kit (Sigma) according to the manufacturer’s instructions. Insoluble fractions of cell lysate were resuspended in 50 mM Tris (pH 7.5) buffer with 8 M urea, centrifuged at 16, 000 × *g*, 4°C for 5 min and re-solubilized insoluble lysate was decanted. Cell lysates were filtered using a 0.45 μm syringe filter to remove solid particulates, then applied to a pre-equilibrated affinity column for purification. The lysate was loaded on a 5 ml Ni-agarose column, which is equilibrated with wash buffer (50 mM Tris-HCl 500 mM NaCl 30 mM imidazole pH 7.5). The column was washed with 10 column volumes of wash buffer and eluted with elution buffer (50 mM Tris-HCl, 500 mM NaCl, 300 mM imidazole, pH 7.5). The eluted protein was passed through a 30 kDa spin column to remove the imidazole. Trehalose was added at a final concentration of 100 mM to this purified protein solution to lyophilize and store the preparation for future use.

Enzyme assay

The activity of chABC was measured by UV-Vis spectrophotometry. The action of chABC generates an 4-5-unsaturated bond in the product. This bond can be monitored at a wavelength of 232 nm. A 4 µg portion of purified enzyme was added to the reaction mixture containing 200 µg of substrate (CS-A/B/C) in 50 mM ammonium acetate at pH 7.4 in a 96-well quartz crystal plate and immediately assayed for chABC activity based on increase in OD_232_ using a Synergy 2 Multimode Microplate Reader (Biotek). Enzyme activity was calculated according to the equation:

$$Enzyme Activity \left( \frac{U}{mL} \right)=\frac{\left( \frac{{\Delta A}_{232}}{\Delta time} \right).V_{total}}{\varepsilon.V_{enzyme}}$$

where 1 U = 1 µmol product formed per min, ΔA_232_ is the change in absorbance over the Δtime, V_total_ is the total volume of the solution, V_enzyme_ is the volume of the enzyme added to the solution and ε is the product disaccharide extinction coefficient (5260 M^−1^ cm^−1^). The commercial standard chABC from *P. vulgaris* was obtained for comparison (AMSBio) and resuspended in sterile water to 20 U/ml.

**Bulk RNA-Seq.** RNA was extracted using the PureLink RNA Mini Kit (Invitrogen) according to manufacturer’s instructions. Two samples were generated from separate scNSC differentiating cultures. scNSCs from three wells of a 12-well plate were pooled for each sample. For **Supplementary Fig. 2c**, single samples were generated from three wells of a 12-well plate for each additional time point in differentiation. RNA-seq was performed at the University of Buffalo Genomics and Bioinformatics Core. Per-cycle basecall (BCL) files generated by the Illumina NextSeq were converted to per-read FASTQ files using bcl2fastq version 2.20.0.422 using default parameters and no lane splitting. The quality of the sequencing was reviewed using FastQC version 0.11.5 and FastqScreen version 0.11.1. Quality reports were summarized using MultiQC version 1.7. No adapter sequences were detected so no trimming was performed. Genomic alignments were performed using hisat2 version 2.1.0 using default parameters. The Illumina provided UCSC hg38 from their igenomes database was used for the reference genome and gene annotation set. Sequence alignments were compressed and sorted into binary alignment map (BAM) files using samtools version 1.7. Counting of mapped reads for genomic features was performed using Subread featureCounts version 1.6.2 using the parameters -p -s 2 –g gene_name -t exon -B -C -Q 60 the annotation file specified with –a was from UCSC hg38. Alignment statistics and feature assignment statistics were again summarized using MultiQC. The unique alignment rate of the paired-end reads for all samples was around 85%. The number of mapped reads assigned to features ranged from about 8 million to 16 million. Differentially expressed genes were2 detected using the Bioconductor package DESeq2 version 1.24.0. DESeq2 tests for differential expression using a negative binomial generalized linear models, dispersion estimates, and logarithmic fold changes. DESeq2 calculates log2 fold changes and Wald test p-values as well as preforming independent filtering and adjusts for multiple testing using the Benjamini-Hochberg procedure to control the false discovery rate (FDR). Histograms of mean data were generated using GrahhPad Prism.

Software:

Bcl2fastq

Illumina. (2018) bcl2fastq Conversion Software. Retrieved August 21, 2018, from https://support.illumina.com/sequencing/sequencing_software/bcl2fastq-conversion-software.html

# FastQC

Babraham Bioinformatics - FastQC A Quality Control tool for High Throughput Sequence Data. (2018) Retrieved August 21, 2018, from http://www.bioinformatics.babraham.ac.uk/projects/fastqc/

# Fastq Screen

https://www.bioinformatics.babraham.ac.uk/projects/fastq_screen/

**Hydrogel biodegradation and solution compatibility.** The primary mode of alginate biodegradation in the mammalian CNS is by diffusion of Ca^2+^ ions out of the gel network^28^. To simulate this, we used a time-course exposure of bulk alginate and hydrogel ribbons to artificial CSF (aCSF) over 21 days as previously described (McKay et al., 2014). For bulk alginate, 200 µl of 1.5% sodium alginate was crosslinked with 100 µl of 100 mM CaCl_2_ in single wells of a 96-well plate followed by 10 min incubation. Three wells (n = 3 repeats) were used for each planned day of measurement (day 1, 3, 5, 7, 10, 14, 21; N = 21 total). On day 0, excess CaCl_2_ was aspirated with a pulled glass Pasteur pipette and the gel rinsed 2X in DI water. Initial wet weight (W_o_) was determined on day 0 and averaged for each condition. Alginate gel was then incubated at 37°C in 200 µl aCSF. aCSF was changed every day to prevent sustained equilibration of Ca^2+^ ions to more accurately mimic the *in vivo* mechanism. Wet weight of the degraded gel (W_f_) on the respective timepoints was used to determine the percent of gel remaining according to the equation: Percent gel remaining = (W_f_/W_o_)*100 (mean ± s.e.m.). n = 3 ribbons for each timepoint were also incubated in aCSF and tested for mechanical integrity by addition of a coverslip to the ribbon on glass slides at corresponding time points for bulk measurements. aCSF was prepared as follows: 119 mM NaCl, 26.2 mM NaHCO_3_, 2.5 mM KCl, 1 mM NaH_2_PO_4_, 1.3 mM MgCl_2_, 10 mM glucose were combined in sterile DI water and gassed for 15 min with 5% CO_2_, followed by the addition 2.5 mM CaCl_2_ (Cold Spring Harbor Labs). For qualitative analysis of ribbon compatibility with numerous commonly used aqueous solutions, 1.5% alginate ribbons were formed in 24-well plates, rinsed 1x in deionized water and resuspended in the solution in question. Samples were incubated at 37°C and observed at regular intervals for dissolution or changes in diameter. **Supplementary Table 5** summarizes gross ribbon behavior in these experiments. Perhaps most notably, ribbons dissolve in high phosphate buffers such as 1x PBS as well as in HBSS or normal saline (0.9%, 154 mM) that lack Ca^2+^ supplementation.

**Visualization of cell-free and cell-laden neural ribbons.** Ribbons were visualized by phase contrast microscopy using the Nikon system described above on glass slides, glass bottom imaging chambers or multi-well plates. Due to ribbon incompatibility with high phosphate buffers, the IF protocol was modified to use 25 mM PIPES/10 mM HEPES as a solvent to prepare ICC buffers in lieu of 1x PBS. Solutions were made fresh and supplemented with 2.5 mM CaCl_2_ to prevent ribbon dissolution. Most often, ribbons were immobilized by gelation of PureCol EZ gel (5 mg/ml; Advanced Biomatrix, #5074) within Nunc Lab-Tek II 4-chambered cover glass (#155382) or Nunc glass bottom dishes with 12 mm viewing areas (#150680). Fluorescein-DHPE, Dextran conjugates or secondary antibodies were mixed into alginate suspensions and co-extruded into 100 mM CaCl_2_ to fluorescently label ribbons. Fluo-4 AM (Invitrogen) dye was added directly to culture medium as per manufacturer’s instructions and diffused into the collagen gel to visualize encapsulated live cells within immobilized ribbons. For fixed samples requiring antibody penetration, immobilizing collagen gel matrix was diluted to 2.5 mg/ml in DMEM/F-12 prior to gelation (37°C, 45 min). **Supplementary Table 2** summarizes fluorescent probes and dyes used in this study, and **Supplementary Table 6** summarizes visualization methods tested in terms of ribbon composition.

**Neural ribbon induction, cell recovery, and differentiation.** scNSC rosettes were generated as previously described above. Dissociated cells were encapsulated at 1 × 10^8^ cells/ml in NovaCol ribbons and cultured in suspension for 5 days in scNSC maintenance medium (N2B27 supplemented with 10 µM SB431542, 2 µM CHIR99021, 200 nM Hh-Ag1.5; 12-well plates)^5^. After 5 days in culture, scNSCs were recovered by dissolving alginate gel in 1.6% sodium citrate and plating onto freshly-coated coverslips for terminal differentiation and IF analysis. Seeded cells were incubated for 15 days additional days in CPM and then transitioned to TDM for SMN maturation. scNSCs from n = 3 replicate ribbons were collected per coverslip. Three repeat experiments were performed (N = 9 total) and averaged. For neurite outgrowth assays, ribbons were transferred to glass bottom dishes and embedded in PureCol EZ gel as previously described. Cultures were maintained in CPM for seven days.

**Supplemental References**

Shaya, D. et al. Composite active site of chondroitin lyase ABC accepting both epimers of uronic acid. *Glycobiology* **18**, 270-277 (2008).

McKay, C. A. et al. An injectable, calcium responsive composite hydrogel for the treatment of acute spinal cord injury. *ACS Appl. Mater.* *Interfaces* **6**, 1424-1438 (2014).
